# Identification of Two Mannosyltransferases Contributing to Biosynthesis of the Fungal-type Galactomannan α-Core-Mannan Structure in *Aspergillus fumigatus*

**DOI:** 10.1038/s41598-018-35059-2

**Published:** 2018-11-16

**Authors:** Takuya Onoue, Yutaka Tanaka, Daisuke Hagiwara, Keisuke Ekino, Akira Watanabe, Kazuyoshi Ohta, Katsuhiko Kamei, Nobuyuki Shibata, Masatoshi Goto, Takuji Oka

**Affiliations:** 1 Department of Applied Microbial Technology, Faculty of Biotechnology and Life Science, Sojo University, Kumamoto, Japan; 2 Department of Infection and Host Defense, Tohoku Medical and Pharmaceutical University, Sendai, Japan; 3 Medical Mycology Research Center, Chiba University, Chiba, Japan; 4 Department of Applied Biochemistry and Food Science, Saga University, Saga, Japan

## Abstract

Fungal-type galactomannan (FTGM) is a polysaccharide composed of α-(1 → 2)-/α-(1 → 6)-mannosyl and β-(1 → 5)-/β-(1 → 6)-galactofuranosyl residues located at the outer cell wall of the human pathogenic fungus *Aspergillus fumigatus*. FTGM contains a linear α-mannan structure called core-mannan composed of 9 or 10 α-(1 → 2)-mannotetraose units jointed by α-(1 → 6)-linkages. However, the enzymes involved in core-mannan biosynthesis remain unknown. We speculated that two putative α-1,2-mannosyltransferase genes in *A. fumigatus*, Afu5g02740/AFUB_051270 (here termed core-mannan synthase A [CmsA]) and Afu5g12160/AFUB_059750 (CmsB) are involved in FTGM core-mannan biosynthesis. We constructed recombinant proteins for CmsA and detected robust mannosyltransferase activity using the chemically synthesized substrate *p*-nitrophenyl α-d-mannopyranoside as an acceptor. Analyses of CmsA enzymatic product revealed that CmsA possesses the capacity to transfer a mannopyranoside to the C-2 position of α-mannose. CmsA could also transfer a mannose residue to α-(1 → 2)-mannobiose and α-(1 → 6)-mannobiose and showed a 31-fold higher specific activity toward α-(1 → 6)-mannobiose than toward α-(1 → 2)-mannobiose. Proton nuclear magnetic resonance (^1^H-NMR) spectroscopy and gel filtration chromatography of isolated FTGM revealed that core-mannan structures were drastically altered and shortened in disruptant *A. fumigatus* strains ∆*cmsA*, ∆*cmsB*, and ∆*cmsA*∆*cmsB*. Disruption of *cmsA* or *cmsB* resulted in severely repressed hyphal extension, abnormal branching hyphae, formation of a balloon structure in hyphae, and decreased conidia formation. The normal wild type core-mannan structure and developmental phenotype were restored by the complementation of *cmsA* and *cmsB* in the corresponding disruptant strains. These findings indicate that both CmsA, an α-1,2-mannosyltransferase, and CmsB, a putative mannosyltransferase, are involved in FTGM biosynthesis.

## Introduction

Galactomannan (GM), consisting of d-mannose (Man) and d-galactofuranose (Gal*f*) residues, is a component of the cell wall surface in filamentous fungi^1–5^. In *Aspergillus fumigatus*, a major pathogenic fungus causing invasive pulmonary aspergillosis, the structure of GM has been elucidated in detail^6,7^. The GM polysaccharide is composed of α-(1 → 2)-/α-(1 → 6)-mannosyl and β-(1 → 5)-/β-(1 → 6)-galactofuranosyl residues and is located at the outermost layer of the *A. fumigatus* cell wall^8^. There are two types of GMs in *A. fumigatus*, fungal-type galactomannan (FTGM) and *O*-mannose-type galactomannan (OMGM)^9^. FTGM contains a linear α-mannan structure called core-mannan in which 9 or 10 α-(1 → 2)-mannotetraose units are concatenated by α-(1 → 6)-linkages^6,7^. Moreover, FTGM also contains galactofuran side chains of β-(1 → 5)-galactofuranotetraose units concatenated by β-(1 → 6)-linkages and bound to the core-mannan by β-(1 → 2), β-(1 → 3), and β-(1 → 6) linkages^6,7^. The FTGM is found in three forms: soluble and released into the extracellular medium, cross-linked to other cell wall polysaccharides, and in membranes anchored to a glycosylphosphatidylinositol^1,3,10^. Part of the FTGM is bound covalently to a branched β-(1 → 3)-glucan^1,11^. Kudoh *et al*. reported that FTGM is attached to the nonreducing terminus of *N*-glycans in *A. fumigatus*^6^. Alternatively, the OMGM consists of β-(1 → 5)-galactofuranosyl chains bound to the nonreducing terminal side of an *O*-mannose-type glycan, where mannosyl chains are attached to a hydroxyl group of serine or threonine in the proteins^6,12^. Like FTGM, the galactofuranosyl residues of OMGM are elongated by β-(1 → 6)-Gal*f*^ 6^. Recently, we reported that GfsA is a β-1,5-galactofuranosyltransferase involved in the biosynthesis of β-(1 → 5)-galactofuranosyl chains, major structures in both FTGM and OMGM^9,13^. However, the enzymes involved in the biosynthesis of core-mannan remain unidentified.

Alpha-1,2-mannosyltransferases have been well studied in the yeast species *Saccharomyces cerevisiae*^14^, *Candida albicans*^15^, and *Schizosaccharomyces pombe*^16^. The *KTR/MNT* mannosyltransferase gene family in the budding yeast *S. cerevisiae* includes *KRE2/MNT1*, *YUR1*, *KTR1*, *KTR2*, *KTR3*, *KTR4*, *KTR5*, *KTR6*, and *KTR7*^14^. The Mnt1/Kre2, Ktr1, and Ktr3 enzymes required for the addition of the second and third mannose residues to *O*-mannose-type glycans in *S. cerevisiae*^14,17^, and Mnt1/Kre2, Ktr1, Ktr2, Ktr3, and Yur1 may be involved in the elaboration of *N*-glycan outer chains^18,19^. The other members Ktr4, Ktr5, and Ktr7 were found to rescue defects in the *C. albicans N*-glycosylation pathway^20^.

We speculated that the *A. fumigatus* homologs of Mnt1/Kre2 include α-1,2-mannosyltransferases involved in core-mannan chain biosynthesis. Based on the Blast algorithm, three putative α-1,2-mannosyltransferase genes were found in the *A. fumigatus* strain Af293/A1163 genome^21^: Afu5g10760/AFUB_058360, Afu5g02740/AFUB_051270, and Afu5g12160/AFUB_059750. The Afu5g10760/AFUB_058360 gene is reported as *afmnt1*, a putative α-1,2-mannosyltransferase involved in cell wall stability and virulence in *A. fumigatus*^21^. However, the functions of Afu5g02740/AFUB_051270 and Afu5g12160/AFUB_059750 have not been characterized.

In this study, we hypothesized that the latter two putative α-1,2-mannosyltransferase genes Afu5g02740/AFUB_051270 and Afu5g12160/AFUB_059750 are involved in biosynthesis of the FTGM core-mannan and are tentatively termed CmsA and CmsB, respectively. To verify this hypothesis, we constructed recombinant CmsA protein for expression in *Escherichia coli* and measured the mannosyltransferase activity. Specific mannosidase analyses of the enzymatic products revealed the CmsA can catalyze the transfer of a mannopyranoside to the C-2 position of α-mannose. Moreover, we demonstrated by proton nuclear magnetic resonance (^1^H-NMR) spectroscopy that CmsA is involved in the biosynthesis of the FTGM core-mannan structure. Finally, we show that disruptant strains ∆*cmsA*, ∆*cmsB*, and ∆*cmsA*∆*cmsB* (∆*cmsAB*) show abnormal growth phenotypes and altered sensitivity to antifungal agents.

## Results

### Features of *cmsA*, *cmsB*, and corresponding protein products in *Aspergillus fumigatus*

**Figure 1:**
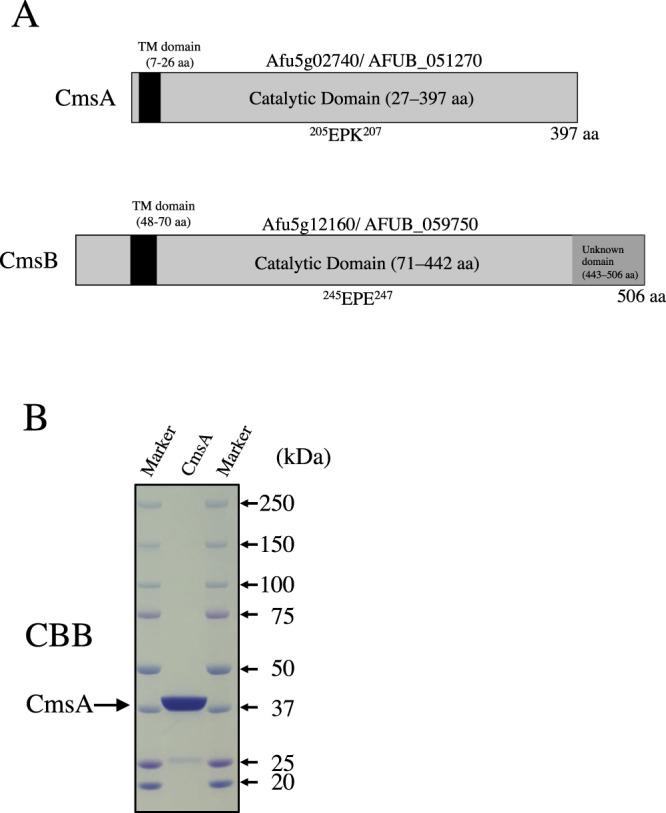
Structure and expression of *A. fumigatus* proteins Afu5g02740/AFUB_051270 (termed core-mannan synthase A, CmsA) and Afu5g12160/AFUB_059750 (termed CmsB). (**A**) Schematic representations of the CmsA and CmsB proteins. The vertical black bars indicate transmembrane (TM) domains of CmsA (7–26 aa) and CmsB (48–70 aa), the gray bars indicate catalytic domains of CmsA (27–397 aa) and CmsB (71–442 aa), and the dark gray bar indicates an unknown domain of CmsB (443–506 aa). ^247^EPD^249^ and ^262^EPN^264^ sequences indicate a DXD-like motif. (**B**) SDS-PAGE analysis of purified recombinant CmsA. Purified recombinant CmsA (4.4 µg) was separated by 5–20% SDS-PAGE and stained with Coomassie brilliant blue, revealing bands of approximately 42 kDa.

The genes Afu5g02740/AFUB_051270 and Afu5g12160/AFUB_059750 are annotated in the Aspergillus genome database (AspGD) as putative transmembrane α-1,2-mannosyltransferases with predicted functions in *N*-linked protein glycosylation^22^. A phylogenetic tree of the three putative α-1,2-mannosyltransferase genes of *A. fumigatus* and the homologous sequences from *S. cerevisiae* and *C. albicans* classified Afu5g02740/AFUB_051270 and Afu5g12160/AFUB_059750 into two gene clusters separate from that containing ScMnt1^21^, suggesting functions distinct from Mnt1. The Afu5g02740/AFUB_051270 and Afu5g12160/AFUB_059750 genes encode predicted 397- and 506-amino acid proteins with molecular masses of 46.5 and 58.9 kDa, respectively (Fig. 1A). TMHMM predicted that both Afu5g02740/AFUB_051270 and Afu5g12160/AFUB_059750 have a transmembrane domain (amino acids 7–26 and 48–70, respectively) at the *N-*terminus, suggesting that these are type II membrane proteins (Fig. 1A)^23^. Several glycosyltransferase families possess a DXD motif (where X represents any amino acid), which is necessary to coordinate the divalent cation^24^. The Mnt1/Kre2 and Ktr4 proteins of *S. cerevisiae* do not contain the archetypal DXD motif^25,26^, but the EPD (amino acids 247–249) and EPN (amino acids 262–264) sequences have been claimed to serve the same purpose^25,26^. The equivalent sequences in CmsA and CmsB were found in EPK (amino acids 205–207) and EPE (amino acids 245–247), as shown in Fig. 1A. The C-terminal side region of Afu5g12160/AFUB_059750 (amino acids 443–506) has no sequence similarity with any previously identified protein domain (Fig. 1A). The Afu5g02740/AFUB_051270 gene is closely related to the genes encoding mannosyltransferases CaKtr4 and ScKtr4 putatively involved in *N*-glycosylation^20,26,27^, while Afu5g12160/AFUB_059750 is closely related to the genes encoding the putative mannosyltransferases ScKtr5 and ScKtr7^20^. We hypothesized that these two putative α-1,2-mannosyltransferase genes are involved in biosynthesis of FTGM core-mannan. Therefore, we tentatively named Afu5g02740/AFUB_051270 core-mannan synthase A (CmsA) and Afu5g12160/AFUB_059750 core-mannan synthase B (CmsB) and attempted to elucidate their functions.

### Enzymatic functions and properties of CmsA

**Figure 2:**
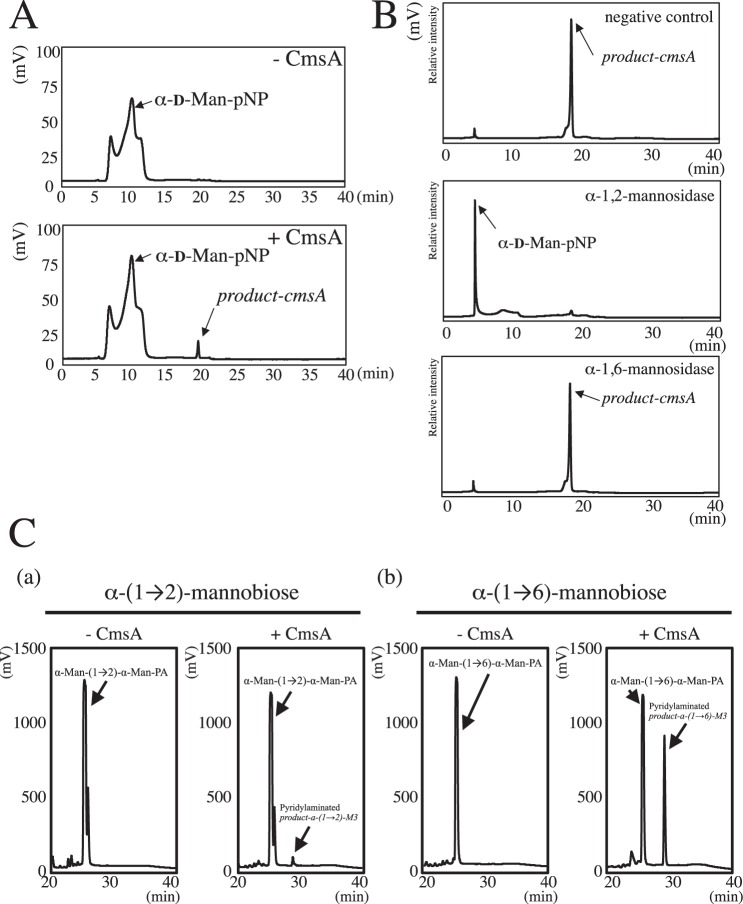
*In vitro* assay of CmsA mannosyltransferase activity. (**A**) Chromatograms of *in vitro* CmsA mannosyltransferase activity assays using *p*-nitrophenyl α-d-mannopyranoside as the substrate. A reaction mixture (20 µl) containing 25 mM HEPES-NaOH (pH 7.0), 50 mM NaCl, 15 mM KCl, 2.5% glycerol, 1.5 mM MnCl_2_, 1.5 mM α-Man-pNP (acceptor), 1.5 mM GDP-Man (donor), and 3.7 µg of purified CmsA was incubated at 37 °C for 24 h. Chromatograms at left show typical results of the assay without CmsA (−CmsA, upper panel) and with CmsA (+CmsA, lower panel). Assays without CmsA yielded only peaks from the substrate (α-Man-pNP) but no other peaks, while fractions with CmsA contained a new reaction product (termed *product-cmsA*) at 18.0 min. (**B**) Determination of *product-cmsA* structure using substrate-specific mannosidases. Upper panels show chromatographs of the purified *product-cmsA*. Purified *product-cmsA* was digested by α-1,2-mannosidase (middle panels) and α-1,6-mannosidase (lower panels). *Product-cmsA* could be digested only by α-1,2-mannosidase and converted to α-Man-pNP. (**C**) Chromatograms of *in vitro* CmsA mannosyltransferase activity assays using α-(1 → 2)-mannobiose (a) or α-(1 → 6)-mannobiose (b) as substrates. A reaction mixture (20 µl) containing 25 mM HEPES-NaOH (pH 7.0), 50 mM NaCl, 15 mM KCl, 2.5% glycerol, 1.5 mM MnCl_2_, 3.7 µg of purified CmsA, 100 mM GDP-Man (donor), and 25 mM α-(1 → 2)-mannobiose (α-Man-(1 → 2)-α-Man) or α-(1 → 6)-mannobiose (α-Man-(1 → 6)-α-Man) (acceptors) was incubated at 30 °C for 60 min. The reaction products were analyzed using HPLC after being labeled with 2-aminopyridine (PA). Chromatograms show typical results from the assay without CmsA (−CmsA, left panel) and with CmsA (+CmsA, right panel). Assays without CmsA yielded only peaks of the substrates (α-Man-(1 → 2)-α-Man-PA or α-Man-(1 → 6)-α-Man-PA) at 25.3 min, whereas those with CmsA exhibited a new reaction product (pyridylaminated *product-a-(1* → *2)-M3* or pyridylaminated *product-a-(1* → *6)-M3*) to which mannose has been transferred and which eluted at 28.9 min.

We obtained recombinant CmsA protein using the *E. coli* expression system. Transcripts encoding the putative catalytic domains of CmsA (amino acids 27–397) and CmsB (amino acids 71–442) were introduced into an *E. coli* expression vector fused in-frame with a 6 × histidine (6 × His) tag at the *N*-terminus. CmsA was successfully expressed as a soluble protein in an *E. coli*, but CmsB was unfortunately expressed as an insoluble protein in an *E. coli*. Recombinant 6 × His-tagged CmsA protein was purified by Ni^+^ affinity chromatography and gel filtration chromatography and analyzed using SDS-PAGE (Fig. 1B). CmsA was visualized as bands of approximately 42 kDa, close to the predicted molecular weights of 45.4 kDa. Next, we measured mannosyltransferase activity at 37 °C using the chemically synthesized substrate *p*-nitrophenyl α-d-mannopyranoside (α-Man-pNP, 1.5 mM) as a sugar acceptor, guanosine diphosphate-α-d-mannose (GDP-Man, 1.5 mM) as a sugar donor, and 1.5 mM Mn^2+^ as a cofactor (Fig. 2A). Reaction mixtures incubated for 24 h without CmsA did not yield any additional peaks on the chromatogram; by contrast, the reaction mixture with CmsA yielded an additional peak at 18.0 min, defined as *peak-cmsA*, suggesting the formation of enzymatic reaction product (defined as *product-cmsA*; Fig. 2A). To determine the chemical structures of *product-cmsA*, we collected and digested the peak fraction using substrate-specific mannosidases (Fig. 2B). *Product-cmsA* was digested by α-(1 → 2)-specific mannosidase but not by α-(1 → 6)-specific mannosidase (Fig. 2B), indicating that *product-cmsA* is α-Man-(1 → 2)-α-Man-pNP. Thus, CmsA has GDP-Man: α-mannoside α-1,2-mannosyltransferase activity *in vitro*. In addition, we attempted to evaluate the elongation activity of CmsA at 30 °C for 60 min using α-(1 → 2)-mannobiose (α-Man-(1 → 2)-α-Man) or α-(1 → 6)-mannobiose (α-Man-(1 → 6)-α-Man) as sugar acceptor substrates (Fig. 2C). The reaction products were analyzed using HPLC after being labeled with 2-aminopyridine (PA). Remarkably, CmsA transferred a Man residue to both α-Man-(1 → 2)-α-Man and α-Man-(1 → 6)-α-Man to generate α-Man-(1 → 2)-α-Man-(1 → 2)-α-Man, and α-Man-(1 → 2)-α-Man-(1 → 6)-α-Man, respectively (Figs 2C and S1). That is because α-(1 → 2)-specific mannosidase digested the pyridylaminated product-α-(1 → 2)-M3 and product-α-(1 → 6)-M3 to generate α-Man-(1 → 2)-α-Man-PA and α-Man-(1 → 6)-α-Man-PA, respectively (Fig. S1). Consistent with the previous report, α-Man-(1 → 2)-α-Man-(1 → 2)-PA was resistant to the α-(1 → 2)-specific mannosidase (Fig. S1)^28^. Specific activities of CmsA toward α-(1 → 6)-mannobiose and α-(1 → 2)-mannobiose were 473 and 15.1 (nmol/min/mg protein), respectively. CmsA showed a 31-fold higher specific activity in the presence of α-(1 → 6)-mannobiose than in that of α-(1 → 2)-mannobiose under the same reaction conditions, indicating that CmsA recognizes α-(1 → 6)-mannobiose as a preferred acceptor substrate rather than α-(1 → 2)-mannobiose (Fig. 2C).

**Figure 3:**
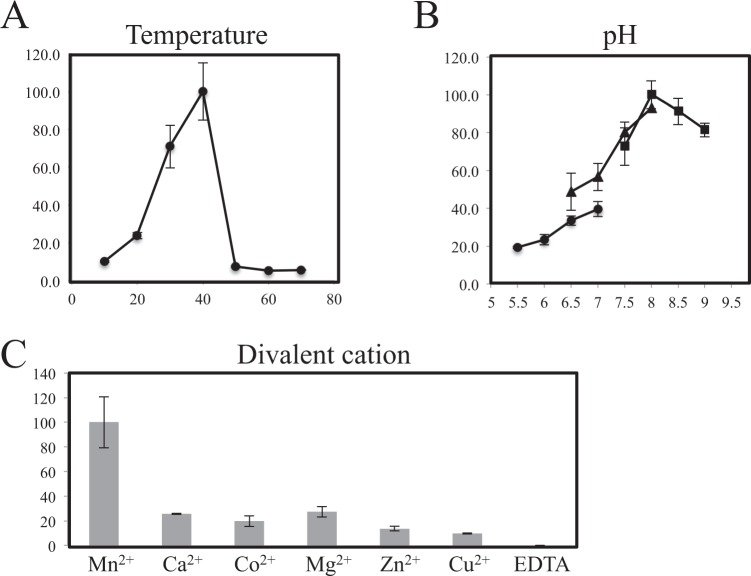
Determination of optimal temperature, pH range, and metal cation requirements for CmsA activity. A reaction mixture (20 µl) containing 20 mM NaCl, 6 mM KCl, 0.1% glycerol, 5 mM MnCl_2_, 10 mM α-Man-pNP (substrate), 10 mM GDP-Man (donor), and 3.7 µg purified CmsA was incubated at 30 °C for 9 h. (**A**) Effect of temperature on CmsA enzymatic activity in 100 mM MOPS-NaOH (pH 7.0). A value of 100% corresponds to the incorporation of 2.21 × 10^−1^ nmol (α-Man-(1 → 2)-α-Man-pNP)/min/mg at 40 °C. (**B**) Effect of pH on CmsA enzymatic activity in 100 mM MES-NaOH (circles), 100 mM MOPS-NaOH (triangles), or 100 mM Tris-HCl (squares). A value of 100% corresponds to the incorporation of 1.32 × 10^−1^ nmol (α-Man-(1 → 2)-α-Man-pNP)/min/mg in 100 mM Tris-HCl at pH 8.0. (**C**) Metal cation requirements for CmsA activity. Reaction mixtures were incubated with 5 mM EDTA or various divalent metals (each 5 mM). A value of 100% corresponds to the incorporation of 6.84 × 10^−1^ nmol (α-Man-(1 → 2)-α-Man-pNP)/min/mg in 5 mM manganese. All data are presented as mean ± SD (*n* = 3 independent experiments).

The divalent metal ions required for CmsA activity were determined using established *in vitro* assays. First, the optimum temperature and pH for the enzyme reaction were determined. The optimum temperature was 40 °C, with activity decreasing sharply at higher temperatures of more than 50 °C (Fig. 3A). The highest enzyme activity was observed in 100 mM Tris-HCl buffer at pH 8.0, with optimum CmsA activity in the range of pH 7.5–8.5 (Fig. 3B). Next, we compared activities using Mn^2+^, Ca^2+^, Co^2+^, Mg^2+^, Zn^2+^, or Cu^2+^ as cofactor and found that Mn^2+^ supported the highest activity although moderate activity was measurable in the presence of Ca^2+^, Co^2+^, or Mg^2+^ (Fig. 3C). Alternatively, no activity was detected in the presence of the divalent cation chelator EDTA. Thus, like many other mannosyltransferases, CmsA requires manganese ions for optimal enzymatic activity, indicating that CmsA belongs to the GT-A superfamily, the members of which typically contain a coordinated divalent cation^29^.

### Deletion of *cmsA*, *cmsB*, or both results in aberrant growth and developmental phenotypes

**Figure 4:**
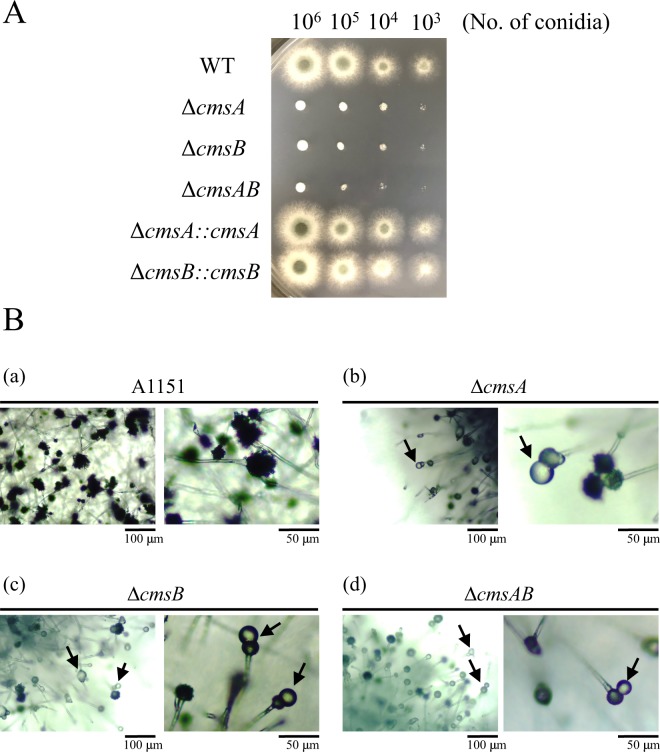
Phenotypic analyses of WT (A1151) and core-mannan synthase disruptants. (**A**) Colony phenotypes of core-mannan synthase disruptants (∆*cmsA*, ∆*cmsB*, ∆*cmsAB*) and corresponding complementary strains (∆*cmsA::cmsA* and ∆*cmsB::cmsB*). Conidia were serially diluted and spotted (number of conidia, 10^6^, 10^5^, 10^4^ and 10^3^). Strains were grown on minimal medium at 37 °C for 36 h. (**B**) Microscopic analysis of WT (A1151), ∆*cmsA*, ∆*cmsB*, and ∆*cmsAB* conidiophores. Conidiophore morphology of WT (A1151) (**a**), ∆*cmsA* (**b**), ∆*cmsB* (**c**) and ∆*cmsAB* (**d**) was observed under 100X (left panels) or 400X (right panels) magnifications. Conidiophores were cultured on minimal medium for 3 days. Scale bars indicate 100 µm (left panels) or 50 µm (right panels). Arrows indicate aberrant conidiophores formed as abnormal swelling structure on the tip or side of vesicles.

**Figure 5:**
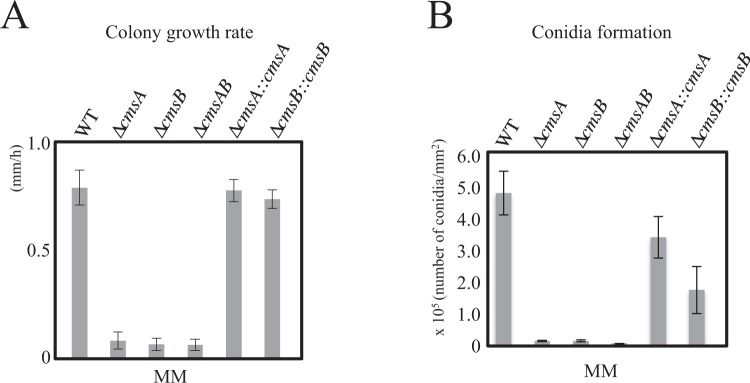
(**A**) Colony growth rates measured. (**B**) Conidia formation. Efficiency of conidiation was analyzed as described in the Materials and Methods.

To elucidate the functions of *cmsA* and *cmsB*, we constructed disruptant strains ∆*cmsA*, ∆*cmsB*, and ∆*cmsA*∆*cmsB* (∆*cmsAB*) (Fig. S2; Table 1) as well as the strains complemented by the corresponding gene, ∆*cmsA::cmsA* for ∆*cmsA* and ∆*cmsB::cmsB for* ∆*cmsB* (Fig. S3; Table 1). The disruptants exhibited impaired growth, hyphal extension, and conidial formation (Figs 4A,B and 5A,B). Strains ∆*cmsA*, ∆*cmsB*, and ∆*cmsAB* formed smaller colonies than the wild type (WT) strain A1151 after cultivation on minimal medium (MM) at 37 °C for 36 h (Fig. 4A), and colony growth rates were markedly reduced (in mm/h, WT: 0.72 ± 0.11 vs. ∆*cmsA*: 0.074 ± 0.03, ∆*cmsB*: 0.058 ± 0.01, ∆*cmsAB*: 0.056 ± 0.02 mm/h; Fig. 5A). ∆*cmsA::cmsA* and ∆*cmsB::cmsB* rescued colony growth rates to near-WT levels (in mm/h, ∆*cmsA::cmsA*: 0.71 ± 0.09, ∆*cmsB::cmsB*: 0.67 ± 0.11) (Fig. 5A).

**Table 1:** *Aspergillus* strains used in this study.

Strains	Genotype	Source of reference
*A. fumigatus* A1151	*KU80::AfpyrG*	da Silva Ferreira, 2006; FGSC
*A. fumigatus* A1160	*KU80::AfpyrG, pyrG* ^−^	da Silva Ferreira, 2006; FGSC
*A. fumigatus ∆glfA*	*KU80::AfpyrG, glfA:: ptrA*	Komachi *et al*.
*A. fumigatus ∆cmsA*	*KU80::AfpyrG, pyrG* ^−^ *, cmsA::AnpyrG*	This study
*A. fumigatus ∆cmsB*	*KU80::AfpyrG, pyrG* ^−^ *, cmsB::AnpyrG*	This study
*A. fumigatus ∆cmsAB*	*KU80::AfpyrG, pyrG* ^−^ *, cmsA::AnpyrG, cmsB::ptrA*	This study
*A. fumigatus ∆cmsA::cmsA*	*KU80::AfpyrG, pyrG* ^−^ *, cmsA::AnpyrG, ∆cmsA::cmsA-ptrA*	This study
*A. fumigatus ∆cmsB::cmsB*	*KU80::AfpyrG, pyrG* ^−^ *, cmsB::AnpyrG, ∆cmsB::cmsB-ptrA*	This study
*A. fumigatus ∆glfA∆cmsA*	*KU80::AfpyrG, pyrG* ^−^ *, cmsA::AnpyrG, glfA:: ptrA*	This study
*A. fumigatus ∆glfA∆cmsB*	*KU80::AfpyrG, pyrG* ^−^ *, cmsB::AnpyrG, glfA:: ptrA*	This study

Conidiophores grown on MM plates at 37 °C for 3 days were then examined by microscopy (Fig. 4B). Conidiophores of strains ∆*cmsA*, ∆*cmsB*, and ∆*cmsAB* were sparse and scattered compared with WT conidiophores (Fig. 4B, left panels). Aberrant conidiophores, which are vesicles without phialide, were observed in all three disruptants (Fig. 4B). Some of the aberrant conidiophores formed the abnormal swelling structure on the tip or side of vesicles of all three disruptants (Fig. 4B). The number of conidia per a conidiophore was lower for all three disruptants compared with that of the WT (Fig. 4B). Indeed, the number of conidia per unit area after 3 days on MM at 37 °C was substantially lower for all three disruptants compared with the WT (in conidia/mm^2^, WT: 4.81 × 10^5^ ± 6.96 × 10^4^, ∆*cmsA*: 1.19 × 10^4^ ± 2.26 × 10^3^ or 2.47% of WT, ∆*cmsB*: 1.21 × 10^4^ ± 3.18 × 10^3^ or 2.51% of WT, ∆*cmsAB*: 4.10 × 10^3^ ± 9.56 × 10^2^ or 0.852% of WT) (Fig. 5B), which was restored in the complementary strains (in conidia/mm^2^, ∆*cmsA::cmsA*: 3.41 × 10^5^ ± 6.60 × 10^4^ and ∆*cmsB::cmsB*: 1.74 × 10^5^ ± 7.47 × 10^4^) (Fig. 5B).

**Figure 6:**
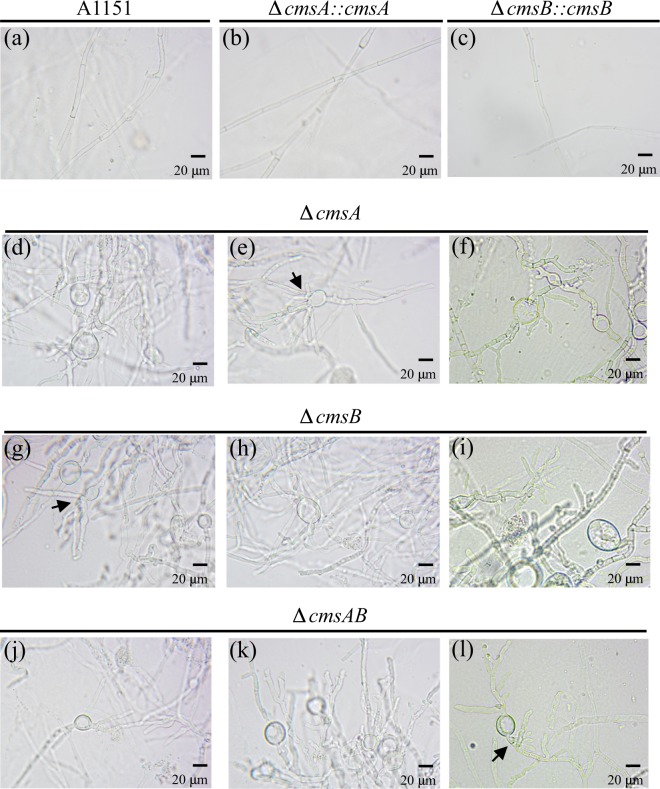
Microscopic analyses of WT (A1151), ∆*cmsA*, ∆*cmsB*, ∆*cmsAB*, ∆*cmsA::cmsA*, and ∆*cmsB::cmsB* hyphae. Mycelial morphology of WT (A1151) (**a**), ∆*cmsA::cmsA* (**b**), ∆*cmsB::cmsB* (**c**), ∆*cmsA* (**d**–**f**), ∆*cmsB* (**g**–**i**) and ∆*cmsAB* (**j**–**l**) under 400X magnification. The mycelia were cultured on minimal medium for 3 days prior to microscope imaging. Scale bars indicate 20 µm. Arrows indicate abnormal branching hyphae.

Structures of the vegetative hyphae were also investigated using microscopy after 3 days on MM at 37 °C (Fig. 6). The hyphae of WT *A. fumigatus* grew linearly, whereas ∆*cmsA*, ∆*cmsB*, and ∆*cmsAB* strains exhibited abnormal swelling (balloon-like structures) and branching (Fig. 6). Taken together, these observations suggest that *cmsA* and *cmsB* are essential for normal hyphae formation and conidiophore development.

### Sensitivity to antifungal agents

Next, we tested the sensitivity of the WT, ∆*cmsA*, ∆*cmsB*, and ∆*cmsAB* strains to the widely used clinical antifungal agents micafungin (MCFG), caspofungin (CPFG), amphotericin B (AMPH-B), flucytosine (5-FC), fluconazole (FLCZ), itraconazole (ITCZ), voriconazole (VRCZ), and miconazole (MCZ) (Table 2). The ∆*cmsA*, ∆*cmsB*, and ∆*cmsAB* strains exhibited only slightly greater sensitivity to AMPH-B, ITCZ, and MCZ compared with the WT strain (Table 2). However, compared with the WT strain, ∆*cmsA* exhibited 8- to 16-fold greater 5-FC sensitivity and ∆*cmsB* exhibited 4-fold greater 5-FC sensitivity (Table 2).

**Table 2:** Sensitivity of the WT, ∆*cmsA*, ∆*cmsB*, and ∆*cmsAB* strains to antifungal agents (µg/mL).

	MCFG	CPFG	AMPH-B	5-FC	FLCZ	ITCZ	VRCZ	MCZ
WT	0.015	0.25	1–2	64	>64	0.5	0.25–0.5	1–2
∆*cmsA*	0.015	0.25	1	4–8	>64	0.25–0.5	0.25–0.5	0.5
∆*cmsB*	0.015	0.25	1	16	>64	0.25	0.25–0.5	0.5
∆*cmsAB*	0.015	0.25	1	4	>64	0.25–0.5	0.5	0.5–1
∆*cmsA::cmsA*	0.015	0.25	1–2	64	>64	1	0.5	2
∆*cmsB::cmsB*	0.015	0.25	1–2	64	>64	0.5	1	2

NOTE. micafungin (MCFG), caspofungin (CPFG), amphotericin B (AMPH-B), flucytosine (5-FC), fluconazole (FLCZ), itraconazole (ITCZ), voriconazole (VRCZ), and miconazole (MCZ).

### Neither CmsA nor CmsB is involved in α-(1 → 2)-mannosyl residue transfer for OMGM biosynthesis *in vivo*

**Figure 7:**
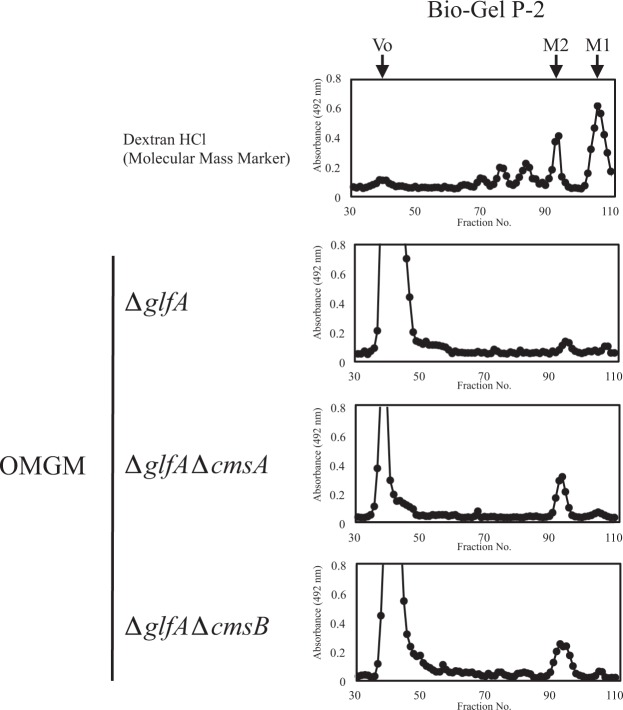
Analyses of OMGM length in ∆*glfA*, ∆*glfA*∆*cmsA* and ∆*glfA*∆*cmsB* strains. OMGMs were prepared from *∆glfA, ∆glfA∆cmsA*, and *∆glfA∆cmsB* strains and analyzed by gel filtration chromatography using a Bio-Gel P-2 (2 × 90 cm) column and dH_2_O as eluent. Arrows indicate elution times of void (Vo), mannose (M1), and mannobiose (M2). The sugar eluted was detected by the phenol-sulfuric acid method. The acid hydrolysis product of dextran was used as a molecular weight marker.

Because α-(1 → 2)-mannosyl residues are present in both FTGM and OMGM^6^, we investigated the influence of *cmsA* and *cmsB* disruption on OMGM structure. Since the presence of β-(1 → 5)-/β-(1 → 6)-galactofuranosyl residues in OMGM prevents the determination of mannosyl chain length, we constructed ∆*glfA*∆*cmsA* and ∆*glfA*∆*cmsB* strains to reveal alterations in the length of mannosyl chain by *cmsA* or *cmsB* disruption (Fig. S4). We extracted total GMs from ∆*glfA*, ∆*glfA*∆*cmsA*, and ∆*glfA*∆*cmsB* strains, released OMGMs by β-elimination treatment, and evaluated the lengths of these released OMGMs by gel filtration chromatography (Bio-Gel P-2 column, 2 × 90 cm, Bio-rad) (Fig. 7) with detection using the phenol-sulfuric acid method. Mannobiose residues were detected in the OMGMs both from ∆*glfA*∆*cmsA* and ∆*glfA*∆*cmsB* strains (Fig. 7), indicating that neither CmsA nor CmsB is involved in the biosynthesis of OMGM α-(1 → 2)-mannosyl residues *in vivo*.

### Involvement of CmsA and CmsB in FTGM core-mannan biosynthesis *in vivo*

**Figure 8:**
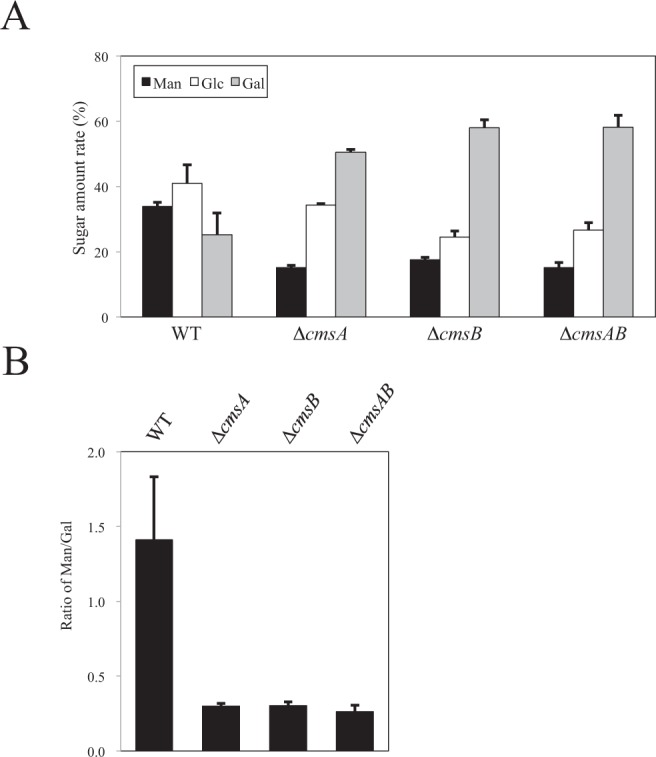
(**A**) Carbohydrate composition of FTGM from strains A1151 (WT), ∆*cmsA*, ∆*cmsB*, and ∆*cmsAB*. All data are presented as mean ± SD (*n* = 3 independent experiments). (**B**) Ratio of mannose/galactose in FTGM obtained from the strains A1151 (WT), ∆*cmsA*, ∆*cmsB*, and ∆*cmsAB*. All data are presented as mean ± SD (*n* = 3 independent experiments).

**Figure 9:**
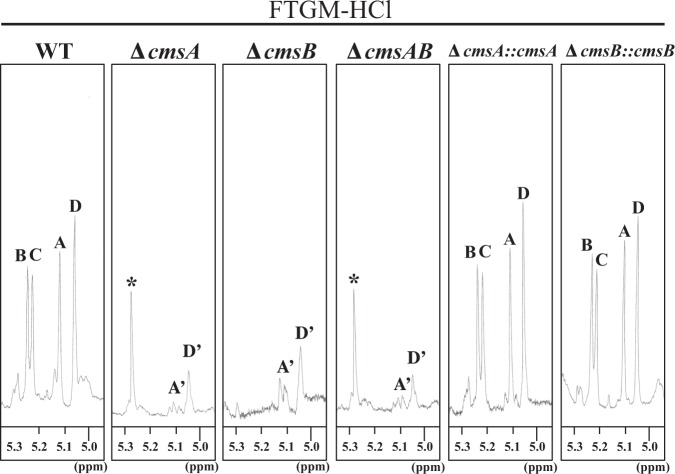
^1^H-NMR analyses of FTGMs without Gal*f* sugar chains (FTGM-HCls) from strains A1151 (WT), ∆*cmsA*, ∆*cmsB*, ∆*cmsAB*, ∆*cmsA::cmsA*, and ∆*cmsB::cmsB*. Total GM (FTGM + OMGM) was extracted and further purified FTGMs were prepared by treatment of GM with 0.5 M NaBH_4_/0.1 M NaOH for 24 h (β-elimination), FTGM-HCl was prepared by acid hydrolysis of the purified FTGMs (0.1 M HCl for 60 min). The signals A (at 5.104 ppm), B (5.233), C (5.216), and D (5.054) of the ^1^H-NMR spectra are derived from H-1 at the C-1 position of the underlined Man residues in the structures -(1 → 6)-α-Man-(1 → 2)-α-Man-(1 → 2)-α-Man-(1 → 2)-α-Man-(1 → 6)- (**A**), -(1 → 6)-α-Man-(1 → 2)-α-Man-(1 → 2)-α-Man-(1 → 2)-α-Man-(1 → 6)- (**B**), -(1 → 6)-α-Man-(1 → 2)-α-Man-(1 → 2)-α-Man-(1 → 2)-α-Man-(1 → 6)- (**C**) and -(1 → 6)-α-Man-(1 → 2)-α-Man-(1 → 2)-α-Man-(1 → 2)-α-Man-(1 → 6)- (**D**). The signals A′ and D′ at 5.1 and 5.05 ppm of the ^1^H-NMR spectra are from H-1 at the C-1 position of the underlined Man residue in t-Man-(1 → 6)-α-Man- and t-Man-(1 → 2)-α-Man-. Asterisks indicate an unidentified NMR signal. The proton chemical shifts were referenced relative to internal acetone at δ 2.225 ppm.

To examine the effects of *cmsA* and *cmsB* gene disruption on FTGM structure, we extracted and purified total GMs from WT A1151, ∆*cmsA*, ∆*cmsB*, ∆*cmsAB*, ∆*cmsA::cmsA*, and ∆*cmsB::cmsB* strains. The extracted total GMs containing both FTGM and OMGM (designated FTGM + OMGM) were then subjected to β-elimination to remove OMGM to designate FTGMs. First, FTGMs were analyzed to determine their carbohydrate compositions (Fig. 8). FTGMs extracted from A1151 (WT-FTGM) were entirely composed of Man (33.8 ± 1.3%), galactose (Gal) (25.3 ± 6.6%), and glucose (40.9 ± 5.8%) residues (Fig. 8A). This result is consistent with previous reports that FTGM is covalently bound to the branched β-(1 → 3)-glucan^1,11^. The calculated Man/Gal ratios in the FTGMs are shown in Fig. 8. The Man/Gal ratios of ∆*cmsA*, ∆*cmsB*, ∆*cmsAB* were noticeably lower than those of A1151 (Fig. 8B). Next, the resultantly purified FTGMs were analyzed using ^1^H-NMR spectroscopy. A strong signal at 5.195 ppm (G in Fig. S5) was detected in ^1^H-NMR spectra from all three disruptant strains. This G peak represents the H-1 at the C-1 position of the underlined Gal*f* residue in -β-Gal*f*-(1 → 5)-β-Galf-(1 → 5)-β-Gal*f*-(1 → 5)- according to a previous report^6^. This result indicates that the Gal*f* sugar chain in FTGM remains even if *cmsA*, *cmsB*, or both genes are disrupted. Thus, to remove galactofuranosyl residues from FTGM, FTGM fractions were subjected to acid hydrolysis and the products (designated FTGM-HCls) analyzed by ^1^H-NMR spectroscopy (Fig. 9). The H-1 signals for the chemical shifts of the α-(1 → 2)-mannan backbone appeared from 5.0 to 5.2 ppm in the ^1^H-NMR spectra. The signals at 5.104 ppm (signal A), 5.233 ppm (signal B), 5.216 ppm (signal C), and 5.054 ppm (signal D) in the WT spectrum (Fig. 9, left panel) represent the H-1 chemical shifts of the underlined Man residues in the structures -(1 → 6)-α-Man-(1 → 2)-α-Man-(1 → 2)-α-Man-(1 → 2)-α-Man-(1 → 6)- (signal A), -(1 → 6)-α-Man-(1 → 2)-α-Man-(1 → 2)-α-Man-(1 → 2)-α-Man-(1 → 6)- (signal B), -(1 → 6)-α-Man-(1 → 2)-α-Man-(1 → 2)-α-Man-(1 → 2)-α-Man-(1 → 6)- (signal C), and -(1 → 6)-α-Man-(1 → 2)-α-Man-(1 → 2)-α-Man-(1 → 2)-α-Man-(1 → 6)- (signal D), respectively, according to a previous report^6^. In contrast, these core-mannan signals were absent or substantially truncated in the ^1^H-NMR spectra for ∆cmsA-FTGM-HCl, ∆cmsB-FTGM-HCl, and ∆cmsAB-FTGM-HCl, indicating that core-mannan structures are altered and/or lost in the absence of *cmsA* or *cmsB* (Fig. 9). A weak signal designated D′ at 5.05 ppm was detected in the ^1^H-NMR spectra for ∆cmsA-FTGM-HCl, ∆cmsB-FTGM-HCl, and ∆cmsAB-FTGM-HCl, while another faint signal designated A′ at 5.1 ppm was detected in the ^1^H-NMR spectra for ∆cmsB-FTGM-HCl and ∆cmsAB-FTGM-HCl (Fig. 9). Signals A′ and D′ are from H-1 at the C-1 position of the underlined Man residue in t-Man-(1 → 6)-α-Man- and t-Man-(1 → 2)-α-Man-^6,30^. Thus, A′ and D′ indicate the presence of α-(1 → 6)-linked and α-(1 → 2)-linked terminal mannosyl residues, respectively, suggesting that shorter oligomannan structures are present in the deletion mutants. The signals A, B, C, and D found in the spectrum from WT-FTGM-HCl were restored in the ^1^H-NMR spectra for the complementary strains ∆*cmsA::cmsA* and ∆*cmsB::cmsB* (Fig. 9). Similar ^1^H-NMR spectra were obtained for FTGMs extracted from strains ∆*glfA*∆*cmsA* and ∆*glfA*∆*cmsB*, in which Gal*f* sugar chains are absent (Fig. S6). These results indicate that core-mannan structures in FTGMs are drastically altered and shortened in the disruptant strains ∆*cmsA*, ∆*cmsB*, and ∆*cmsAB*.

**Figure 10:**
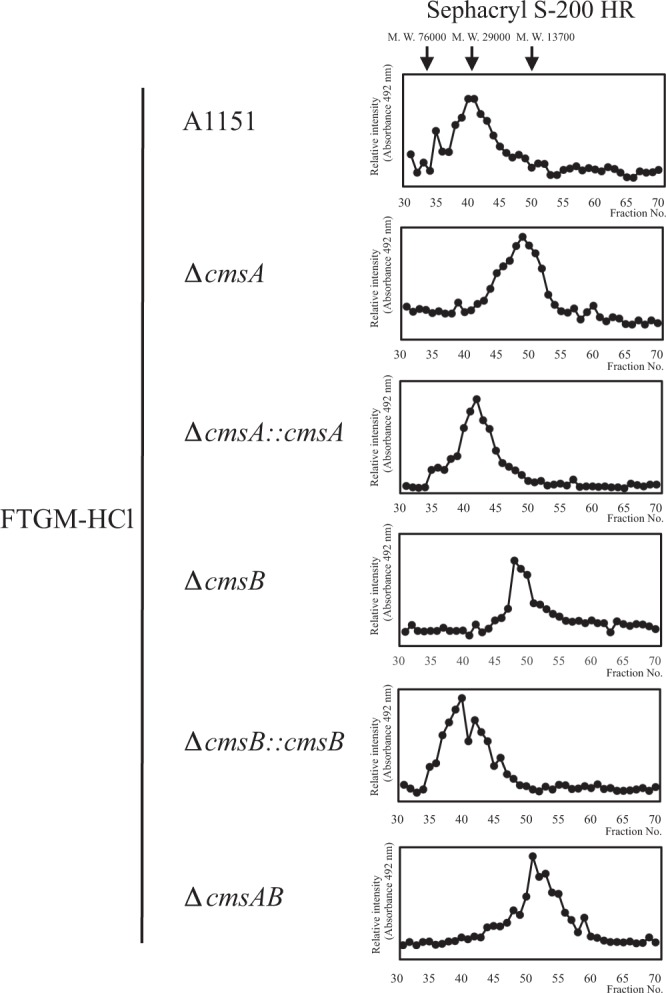
Gel filtration analyses of FTGM-HCls from strains A1151 (WT), ∆*cmsA*, ∆*cmsB*, ∆*cmsAB*, ∆*cmsA::cmsA*, and ∆*cmsB::cmsB*. FTGM-HCls were prepared and analyzed by gel filtration chromatogyraphy using a Sephacryl S-200 HR (1 × 75 cm) column and 0.8 M NaCl for elution.

Next, these FTGM-HCls were analyzed by gel filtration chromatography using a Sephacryl S-200 HR column (1 × 75 cm) and detection by the phenol-sulfuric acid method. Peak FTGM-HCl content in the WT A1151 strain was detected from fractions 35 to 50 (Fig. 10). In ∆*cmsA*, ∆*cmsB*, and ∆*cmsAB*, the molecular weights of FTGM-HCls were shifted downward (Fig. 10), while the gene complemented strains ∆*cmsA::cmsA* and ∆*cmsB::cmsB* restored the molecular masses of FTGM-HCls similar to those of WT (Fig. 10). Similar chromatograms were obtained for FTGMs extracted from strains ∆*glfA*∆*cmsA* and ∆*glfA*∆*cmsB* (Fig. S7). These results indicate that the average molecular weights of FTGM-HCl are reduced in the absence of *cmsA* and *cmsB*. Taken together, these results indicate that CmsA is an α-1,2-mannosyltransferase that is responsible for FTGM core-mannan biosynthesis and CmsB is a putative α-1,2-mannosyltransferase responsible for FTGM core-mannan biosynthesis.

## Discussion

We elucidated that CmsA has α-1,2-mannosyltransferase using *in vitro* mannosyltransferase assay and substrate-specific-mannosidase analysis (Figs 2 and S1). On the contrary, we could not obtain soluble CmsB recombinant proteins using *E. coli* expression systems. CmsB protein has 42% amino acid sequence identities with an α-1,2-mannosyltransferase, ScKtr7 protein and 37.5% amino acid sequence identities with CmsA. Therefore, it is speculated that CmsB also has α-1,2-mannosyltransferase activity *in vitro* and *in vivo*. However, detailed analyses will be required for the identification of the enzymatic function of CmsB.

^1^H-NMR spectroscopy and gel filtration chromatography revealed that CmsA and CmsB are required for the biosynthesis of FTGM core-mannan (Figs 9, 10 and S6, S7). Because the phenotypes of ∆*cmsA* are similar to those of ∆*cmsB*, these enzymatic functions may not be redundant. Therefore, it is believed that CmsA and CmsB are involved in the transfer of mannose residues at different positions in FTGM. Because glycans are sequentially biosynthesized, CmsB is likely involved in mannose transfer during the early stages of FTGM biosynthesis. CmsA demonstrated weaker elongation activity toward α-(1 → 2)-mannobiose than toward α-(1 → 6)-mannobiose *in vitro* (Fig. 2C). The result indicates that in the FTGM structure, CmsA is responsible for transferring the mannose to the C-2 position of the α-mannose residue at the non-reducing side of α-(1 → 6)-mannobiose. Further, the possibility that CmsB primarily acts as an elongation enzyme of α-(1 → 2)-mannobiose *in vivo* cannot be excluded. However, functional differences between CmsA and CmsB are unknown, necessitating further detailed analysis. Nonetheless, there is no doubt that CmsA and CmsB are involved in the biosynthesis of the FTGM core-mannan. Notably, β-(1 → 5)-galactofuranosyl chains were retained in FTGM (β-eliminated GM) extracted from ∆*cmsA* and ∆*cmsB*, indicating that not all β-(1 → 5)-galactofuranosyl chains are bound to the core-mannans of FTGM and OMGM (Fig. S5). It is conceivable that some β-(1 → 5)-galactofuranosyl chains are bound to unknown sugar chain structures.

Wagener *et al*. reported that *afmnt1* (Afu5g10760/AFUB_058360 gene) is a putative α-1,2-mannosyltransferase and is required for cell wall stability^21^. A ∆*afmnt1* strain exhibited increased sensitivity to cell wall stress, high temperature, and calcofluor white and Congo red staining^21^. However, the ∆*afmnt1* mutant normally grows under normal growth conditions^21^. In contrast, both ∆*cmsA* and ∆*cmsB* abnormally grow, and their growth defects are considerable. This observation suggests that the function of AfMnt1 is different than that of both CmsA and CmsB. However, the enzymatic function(s) of AfMnt1 have yet to be elucidated, necessitating further detailed analysis. Recently, a multiple deletion mutant of 11 putative mannosyltransferases other than *cmsA* and *cmsB* that is responsible for establishing α-(1 → 6)- and α-(1 → 2)-mannose linkages in yeast was constructed in *A. fumigatus*^31^. The mutant did not reduce the mannan content of the mycelium cell wall but reduced the mannan content of the conidial cell wall^31^. The mutant did not exhibit drastic mycelial growth defects^31^. Therefore, core-mannan constructed by CmsA and CmsB seemingly plays an important role in mycelial cell wall maintenance rather than these 11 mannosyltransferases.

Orthologs of *cmsA* and *cmsB* are also present in many pathogenic fungi that cause plant and animal diseases, such as the animal pathogens *Trichophyton rubrum*, *Paracoccidioides brasiliensis*, and *Ajellomyces capsulatus* and the plant pathogens *Magnaporthe grisea*, *Fusarium graminearum*, and *Botrytis cinerea*. There are a few previous reports on phenotypic changes conferred by disruption of *cmsA* and/or *cmsB* orthologs. In *Neurospora crassa*, strong growth inhibition was observed by disruption of the *cmsA* ortholog^32^, suggesting common functions for CmsA in filamentous fungi. However, in the insect pathogenic fungus *Beauveria bassiana*, disruption of the *cmsA* ortholog *ktr4* or *cmsB* ortholog *ktr1* did not cause drastic growth inhibition^33^, suggesting that *cmsA* and *cmsB* have different functions in *B. bassiana* or that core-mannan elaborated by CmsA and CmsB does not play an important role in *B. bassiana* development.

Loss of the core-mannan chain was associated with abnormal structures of hyphae and conidiophore as well as reduced conidia formation, indicating that the core-mannan chain plays an important role in the normal formation of hyphal and conidiophore cell walls (Figs 4–6). Reportedly, similar phenotypes are also observed in disrupted strains of the genes involved in the biosynthesis of GDP-Man, a sugar donor of CmsA and CmsB^34–36^. A similar balloon structure was also reported in other cell wall-deficient strains, for example, strains missing chitin-synthase with myosin domain (*csmA*)^37^, protein O-mannosyltransferase A and C (*pmtA* and *pmtC*)^38–40^. Given that core-mannan deficiency decreases the physical strength of the cell wall, strains ∆*cmsA* and ∆*cmsB* may not be able to withstand the osmotic pressure of the external environment, resulting in formation of the observed balloon structure. Disruption of *cmsA* and *cmsB* also repressed hyphae extension, suggesting that CmsA and/or CmsB inhibitors may be effective antifungal drugs. FTGM is a sugar chain structure specific to fungi and does not exist in humans or plants (that do not possess homologous enzymes). Thus, we assume that the α-1,2-mannosyltransferases involved in FTGM biosynthesis are potential targets for use in novel antifungal therapies. Moreover, *cmsA* and/or *cmsB* disruptant strains demonstrated increased susceptibility to several clinical antifungal drugs (AMPH-B, ITCZ, MCZ, and 5-FC), suggesting that CmsA and/or CmsB inhibitors may enhance the therapeutic efficacy of these compounds (Table 2).

In conclusion, the deletion of CmsA, α-1,2-mannosyltransferase, and/or CmsB, putative α-1,2-mannosyltransferase involved in FTGM biosynthesis, induces severe developmental impairments and increases susceptibility to several existing antifungal drugs. This suggests the possibility of developing new drugs targeting α-1,2-mannosyltransferase to combat fungal pathogens in medicine and agriculture.

## Materials and Methods

### Microorganisms and growth conditions

The *A. fumigatus* strains used in this study are listed in Table 1. *Aspergillus fumigatus* strains A1160 and A1151 were obtained from the Fungal Genetics Stock Center (Kansas City, MO) and grown on MM composed of 1% glucose, 0.6% NaNO_3_, 0.052% KCl, 0.052% MgSO_4_·7H_2_O, and 0.152% KH_2_PO_4_ plus Hunter’s trace elements (in w/v; pH 6.5). *Aspergillus* strains were transformed using standard procedures. Plasmids were amplified in *E. coli* DH5α and proteins expressed in *E. coli* strain Rosetta-gami B (DE3) (Merck Millipore, Darmstadt, Germany).

### Construction of expression vectors for CmsA and CmsB

Bacterial codon-optimized *cmsA* (AFUB_051270) and *cmsB* (AFUB_059750) genes from *A. fumigatus* A1163 were synthesized by Bioneer (Korea). The *cmsA* gene was delivered by Bioneer cloned in a pBT7-N-His vector with an N-terminal 6 × His tag, termed pBT7-CmsA. The pBT7-CmsA was used for recombinant protein production. The *cmsB* gene was amplified by PCR and cloned into pET15b-KAI^9^. The *cmsB* gene was amplified by PCR using the cloned synthesized *cmsB* gene as a template for the primers pET15b-CmsB(CO)-IF-F and pET15b-CmsB(CO)-IF-R (Table S1). The amplified fragment was inserted between the *Nde* I and *Not* I sites of pET15b-KAI by in-fusion HD cloning kit (TAKARA, Japan) to yield pET15b-CmsB. All PCR reactions were performed using Phusion High-Fidelity DNA Polymerase (New England Biolabs, USA). The pBT7-CmsA and pET15b-CmsB plasmids were transformed into Rosetta-gami B (DE3) cells.

### Protein purification, quantification, and electrophoresis

Protein purification was performed as previously described^9^. Protein concentrations were determined using the Qubit Protein Assay Kit (Thermo Fisher Scientific, USA), and CmsA and CmsB were analyzed by SDS-PAGE to assess purity and molecular weight.

### Enzyme assays

The artificial acceptor substrate *p*-nitrophenyl α-d-mannopyranoside (α-Man-pNP) was purchased from Sigma-Aldrich (USA). Alpha-(1 → 2)-mannobiose and α-(1 → 6)-mannobiose were purchased from Dextra Laboratories Ltd (United Kingdom). Standard assays were performed with α-Man-pNP, α-(1 → 2)-mannobiose or α-(1 → 6)-mannobiose as acceptor, GDP-Man as donor, and purified CmsA protein in a total reaction volume of 20 µl. The mixture was incubated at 37 °C and stopped by heating (99 °C) for 5 min. Para-nitrophenol derivatives were detected by UV_300_ absorbance and analyzed by HPLC using a Shodex Asahipak NH2P-50 4E amino column (250 × 4.6 mm; Showa Denko, Japan) as previously described^9^. Alpha-(1 → 2)-mannobiose or α-(1 → 6)-mannobiose derivatives were labeled using 2-aminopyridine (PA) after reaction as previously described, with slight modifications^41^. Reaction mixtures were dried using a centrifugal concentrator, and the resulting samples were dissolved in 20 µL of PA reagent (110.4 mg of PA in 40 µL of glacial acetic acid) and incubated at 90 °C for 1 h. The samples were then mixed with 70 µL of PA reducing reagent (200 mg borate–dimethylamine complex in 80 µL of glacial acetic acid and 50 µL of ultra-pure water) and incubated at 80 °C for 35 min. Next, 500 µL of 0.2 M ammonia solution and 300 µL of chloroform were added to the resulting sample. Following centrifugation, the supernatant was designated as the PA-sugars. The PA-sugars were purified with a Monospin PBA spin column (GL Science, Tokyo, Japan) as previously described^42^. The purified PA-sugars were detected via fluorescence at excitation and emission wavelengths of 310 nm and 380 nm, respectively, and analyzed using HPLC with a Shodex Asahipak NH2P-50 4E amino column (250 × 4.6 mm) as follows. Elution was performed using two solvents: eluent A, 93% acetonitrile in 0.3% acetate (pH adjusted to 7.0 with ammonia), and eluent B, 20% acetonitrile in 0.3% acetate (pH adjusted to 7.0 with ammonia). The gradient program was set at a flow rate of 0.8 mL/min (expressed as the percentage of solvent B): 0–5 min, isocratic 3%; 5–30 min, 3–40%; 30–35 min, 40–3%; 35–60 min, isocratic 3%. Enzymatic activity was evaluated by determining the area of products *via* HPLC. Oligosaccharide levels were compared to α-Man-pNP, α-(1 → 2)-mannobiose or α-(1 → 6)-mannobiose as a standard. Alpha-1,2-mannosidase and α-1,6-mannosidase were purchased from ProZyme (USA) and New England Biolabs (USA), respectively, and used according to the manufacturers’ instructions.

### Construction of ∆*cmsA* and ∆*cmsB* gene disruption strains

*A. fumigatus* A1160 was used as the parental strain (Table 1) to construct *∆cmsA* and *∆cmsB* strains by *AnpyrG* insertion (Fig. S2A). DNA fragments for the gene disruption were constructed using a “double-joint” PCR method as previously described^43^. All PCRs were performed using Phusion High-Fidelity DNA Polymerase and the oligonucleotide primers listed in Table S1. Eight primers, AFUB_051270–1 to AFUB_051270-4, AFUB_051270-7, AFUB_051270-8, pyrG-5, and pyrG-6, were used to construct a deletion cassette for ∆*cmsA*. Similarly, eight primers, AFUB_059750-1 to AFUB_059750-4, AFUB_059750-7, AFUB_059750-8, pyrG-5, and pyrG-6, were used to construct a deletion cassette for ∆*cmsB*. The 5′- and 3′-flanking regions (approximately 1.0 kb each) of *cmsA* were PCR amplified from genomic DNA with primer pairs AFUB_051270-1/AFUB_051270-2 and AFUB_051270-3/AFUB_051270-4. The 5′- and 3′-flanking regions (approximately 1.0 kb each) of *cmsB* were PCR amplified from genomic DNA with primer pairs AFUB_059750-1/AFUB_059750-2 and AFUB_059750-3/AFUB_059750-4. The *AnpyrG* gene used as a selective marker was amplified using plasmids pSH1 as template^13^ and the primer pairs pyrG-5/pyrG-6. The three amplified fragments were purified, mixed, and subjected to a second PCR without specific primers to assemble each fragment as the overhanging chimeric extensions act as primers. A third PCR was performed with the nested primer pairs AFUB_051270-7/AFUB_051270-8 for *cmsA* or AFUB_059750-7/AFUB_059750-8 for *cmsB* and the products of the second PCR used as templates to generate the final deletion constructs. The amplified final deletion constructs were purified using the Fast Gene Gel/PCR Extraction Kit (NIPPON GENE, Japan) and used directly for transformation of *A. fumigatus*. Transformants were grown on MM plates containing 0.6 M KCl as an osmotic stabilizer under appropriate selection conditions, and single colonies were isolated twice before further analysis. Correct replacement of the DNA fragments for gene complementation was confirmed by PCR using the primer pairs AFUB_051270-1/pyrG-R and pyrG-F/AFUB_051270-4 for *cmsA* and the primer pairs AFUB_059750-1/pyrG-R and pyrG-F/AFUB_059750-4 for *cmsB* (Fig. S2B). At least three of these mutants were independently constructed.

### Construction of the ∆*cmsA*∆*cmsB* (∆*cmsAB*) double gene disruption strain

*A. fumigatus* ∆*cmsA* was used as the parental strain (Table 1) to construct the *cmsA and cmsB* double gene disruption strain (***∆****cmsAB*). The *cmsB* gene was disrupted in *A. fumigatus* ∆*cmsA* strain by *ptrA* insertion (Fig. S2A). The 5′- and 3′-flanking regions (approximately 1.0 kb each) of *cmsB* were PCR amplified from genomic DNA with primer pairs AFUB_059750-1/AFUB_059750-2P and AFUB_059750-3P/AFUB_059750-4. The *ptrA* gene used as a selective marker was amplified using plasmid pPTR-I (Takara) as template and the primer pair ptrA-5/ptrA-6. Disruption of *cmsB* was confirmed by PCR using the primer pairs AFUB_059750-1/ptrA-R and ptrA-F/AFUB_059750-4 (Fig. S2B). At least three of these mutants were independently constructed.

### Construction of ∆*cmsA* and ∆*cmsB* complementary strains (∆*cmsA::cmsA* and ∆*cmsB::cmsB*)

*A. fumigatus* ∆*cmsA* and ∆*cmsB* were used as the host strains (Table 1) for constructing the complemented strains (Fig. S3A). All PCR assays were performed using Phusion High-Fidelity DNA Polymerase. Eight primers (AFUB_051270-1, AFUB_051270-complement-2, complement-3, complement-4, complement-8, AFUB_051270-7, ptrA-F, and ptrA-R) were used to construct a complementation cassette for *cmsA* and eight primers (AFUB_059750-1, AFUB_059750-complement-2, complement-3, complement-4, complement-8, AFUB_059750-7, ptrA-F, and ptrA-R) to construct a complementation cassette for *cmsB*. The regions of the *cmsA* and *cmsB* gene were PCR amplified from genomic DNA using the primer pairs AFUB_051270-1/AFUB_051270-complement-2 and AFUB_059750-1/AFUB_059750-complement-2, respectively. The region of the *pyrG* gene from *A. nidulans* (*AnpyrG*) was PCR amplified from pSH1 using the primer pair complement-3/complement-4. The *ptrA* genes used as selective markers were amplified using the pPTR-I plasmid as a template and the primer pair ptrA-5/ptrA-6. The three amplified fragments were purified, mixed, and subjected to a second PCR assay without specific primers to assemble each fragment as the overhanging chimeric extensions acted as primers. A third PCR assay was performed using the products of the second PCR as a template and the nested primer pair AFUB_051270-7/complement-8 for *cmsA* and AFUB_059750-7/complement-8 for *cmsB* to generate the final DNA constructs. The amplified final deletion constructs were purified using the Fast Gene Gel/PCR Extraction Kit and used directly for transformation. Transformants were grown on MM plates containing 0.6 M KCl as an osmotic stabilizer under appropriate selection conditions, and single colonies were isolated twice before further analysis. Correct replacement of the DNA fragments for gene complementation was confirmed by PCR using the primer pairs AFUB_051270-1/ptrA-R and ptrA-F/AFUB_051270-4 for *cmsA* and AFUB_059750-1/ptrA-R and ptrA-F/AFUB_059750-4 for *cmsB* (Fig. S3B).

### Construction of ∆*glfA*∆*cmsA* and ∆*glfA*∆*cmsB* strains

*A. fumigatus* ∆*cmsA* and ∆*cmsB* were used as host strains (Table 1) to construct ∆*glfA*∆*cmsA and* ∆*glfA*∆*cmsB strains*. The *glfA* gene was disrupted in *A. fumigatus* ∆*cmsA* and ∆*cmsB* strains by *ptrA* insertion (Fig. S4). The DNA fragment containing the *ptrA* marker for disruption of *glfA* was amplified by PCR from the genomic DNA of ∆*glfA* using the primer pair AFUB_036480-7/AFUB_036480-8^13^. Disruption of target genes was confirmed by PCR using the primer pairs AFUB_036480-1/ptrA-R and ptrA-F/AFUB_036480-4 (Fig. S4).

### Preparation of FTGM

Preparation of total GM (FTGM + OMGM) from *A. fumigatus* was performed as previously described^9^. The cell wall extract was fractionated by cetyl trimethyl ammonium bromide (CTAB) using a previously described method^44^. A CTAB fraction precipitated at pH 9.0 with NaOH in the presence of borate was recovered as the galactomannoprotein fraction. A β-elimination reaction was performed by exposing the fractionated FTGM + OMGM to reducing alkali conditions (0.5 M NaBH_4_/0.1 M NaOH, 10 ml, at 25 °C for 24 h)^30^. After neutralized with 50% acetic acid solution, resultant samples were dialyzed overnight against distilled water. The purified samples were then lyophilized, resuspended in distilled water, and clarified using 0.45-µm pore filters. These β-eliminated FTGM + OMGMs were designated the purified FTGMs^9^.

### Preparation of FTGM-HCls

To remove the Gal*f* sugar chains, FTGMs were treated with 0.1 M hydrochloric acid at 100 °C for 60 min. Samples were then neutralized with 10 M NaOH and dialyzed overnight against dH_2_O^6^. These acid-treated FTGMs, designated FTGM-HCls, contained only core-mannan without Gal*f* sugar chains.

### Preparation of OMGM

To prepare OMGM, a β-elimination reaction was performed against the fractionated FTGM + OMGM under nonreducing alkali conditions (0.1 M NaOH, 10 ml, at 25 °C for 24 h). Samples were neutralized with 50% acetic acid solution, lyophilized, resuspended in dH_2_O, and clarified using 0.45-µm pore filters. These samples were designated as OMGMs.

### Gel filtration chromatography of FTGM-HCl

Separation of FTGM-HCls by gel filtration chromatography was conducted using a method described previously with minor modifications^6^. Briefly, 5 mg FTGM-HCls were applied to the column at 0.5 ml/min. Gel filtration chromatography was performed using a Sephacryl S-200 HR (1 × 75 cm) column (GE Healthcare, USA) with 0.8 M NaCl as eluent. One hundred 1-ml fractions were collected. The eluted sugars were monitored using phenol-sulfuric acid method^45^.

### Gel filtration chromatography of OMGMs

OMGMs were separated by gel chromatography using a previously described method with minor modifications^6^. Briefly, 200 mg OMGMs were applied to a Bio-Gel P-2 (2 × 90 cm) column (Bio-Rad Laboratories, Hercules, CA, USA) at 0.5 ml/min and eluted using dH_2_O. One hundred 5-ml fractions were collected. The eluted sugar chains were monitored using the phenol-sulfuric acid reaction^45^.

### Carbohydrate composition analysis

Carbohydrate composition analysis was performed as described previously, with slight modifications^6^. Briefly, 1 mg of each FTGM was hydrolyzed with 4 M trifluoroacetic acid (TFA) at 110 °C for 3 h. After removal of the TFA via evaporation, resulting monosaccharides were reduced overnight with 1% NaBH_4_ at room temperature. The resulting alditols were acetylated with pyridine/acetic anhydride (1:1, v/v) at 40 °C for 12 h. The reagents were then removed via evaporation with toluene, dissolved in chloroform, and washed thrice with water. The resulting alditol acetate derivatives were analyzed via gas chromatography-mass spectrometry (GC-MS) using a capillary column (30 m × 0.32 mm; DB-5, Agilent Technologies, USA) with helium as the carrier gas and a temperature program of 160 °C–200 °C at 3 °C/min. The GC-MS analyses were performed using the JEOL JMS-K9 mass spectrometer (Japan).

### Nuclear Magnetic Resonance spectroscopy

Samples for NMR were exchanged twice in D_2_O with intervening lyophilization and were then dissolved in D_2_O (99.97% atom ^2^H). The NMR spectra were recorded using a JNM-LA600 spectrometer (JEOL) at 45 °C. The proton and carbon chemical shifts were referenced relative to internal acetone at δ 2.225 and 31.07 ppm, respectively.

### Colony growth rate determination

Colony growth rates were measured as described previously^13^. Briefly, conidia from each strain were point inoculated into the center of agar plates and colony diameters measured after 24, 48, 72, 96, and 120 h of incubation at 37 °C. The growth rates were determined for each colony in millimeters per hour during each of the incubation intervals (24–48, 48–72, 72–96, and 96–120 h) and then averaged across the entire time interval. Growth rate measurements were repeated 20 times for each individual strain.

### Analysis of conidiation efficiency

The efficiency of conidiation was analyzed as described previously^13^. Briefly, approximately 10^5^ conidia were spread onto MM plates (90 mm diameter). After 3 days of incubation at 37 °C, the conidia were suspended in 15 ml of 0.01% (w/v) Tween 20 and counted using a hemocytometer. Efficiency was quantified as the number of conidia formed per unit area.

### Drug susceptibility testing

Drug susceptibility was tested in triplicate according to the Clinical and Laboratory Standards Institute reference broth microdilution method (document M38-A2)^46^, with minor modifications. Briefly, dried plates were used for antifungal susceptibility testing (Eiken Chemicals, Japan)^47^.

### Software and database searches

The transmembrane regions of CmsA and CmsB were predicted by the TMHMM tool (http://www.cbs.dtu.dk/services/TMHMM/)^23^. BLAST searches were performed by a tool in Aspergillus genome database (AspGD) (http://www.aspergillusgenome.org)^22^.

## Electronic supplementary material

Supplementary-materials
