# Author Correction: Identification of Two Mannosyltransferases Contributing to Biosynthesis of the Fungal-type Galactomannan α-Core-Mannan Structure in *Aspergillus fumigatus*

**DOI:** 10.1038/s41598-020-67911-9

**Published:** 2020-07-09

**Authors:** Takuya Onoue, Yutaka Tanaka, Daisuke Hagiwara, Keisuke Ekino, Akira Watanabe, Kazuyoshi Ohta, Katsuhiko Kamei, Nobuyuki Shibata, Masatoshi Goto, Takuji Oka

**Affiliations:** 1 Department of Applied Microbial Technology, Faculty of Biotechnology and Life Science, Sojo University, Kumamoto, Japan; 2 Department of Infection and Host Defense, Tohoku Medical and Pharmaceutical University, Sendai, Japan; 3 Medical Mycology Research Center, Chiba University, Chiba, Japan; 4 Department of Applied Biochemistry and Food Science, Saga University, Saga, Japan

Correction to: *Scientific Reports* 10.1038/s41598-018-35059-2, published online 16 November 2018

In this Article Table S1 was omitted from the Supplementary Information file. Table S1 can be seen below.

**Table S1:** Oligonucleotides used in this study

Oligonucleotide primers	Sequence
AFUB_051270-1	5′-CTTCGGAGGCAATGGGACTG-3′
AFUB_051270-2 (*cmsA:: AnpyrG*)	5′-GGCGTGATAGCGTTGAAGGGCGATAACCGATAGCCAGAGGG-3′
AFUB_051270-3 (*cmsA:: AnpyrG*)	5′-AGGTTCCTTTGTGGCTGGGACCAAGGAAGACTGCAGACCC-3′
AFUB_051270-4	5′-TCGCTTATGATCTCGTCCCC-3′
AFUB_051270-7	5′-TAGTGTCTGTGGGATTGGGG-3′
AFUB_051270-8	5′-AGGTCCTCTCAGCTGCTCT-3′
AFUB_059750-1	5′-TTGACCCTGCATGGAGAGTG-3′
AFUB_059750-2 (*cmsB:: AnpyrG*)	5′-GGCGTGATAGCGTTGAAGGGGCTGCGTCGACTTGTTTGAA-3′
AFUB_059750-3 (*cmsB:: AnpyrG*)	5′-AGGTTCCTTTGTGGCTGGGAGGAGGATGGGCATCTCCATA-3′-3′
AFUB_059750-2P (*cmsB::ptrA*)	5′-ATGCAAGAGCGGCTCATCGGCTGCGTCGACTTGTTTGAA-3′
AFUB_059750-3P (*cmsB::ptrA*)	5′-TCGTTACCAATGGGATCCCGGAGGATGGGCATCTCCATA-3′
AFUB_059750-4	5′-CGTCTCTGGAAGGAAGTGCT-3′
AFUB_059750-7	5′-GCGTACTCCAGGAGGTACGT-3′
AFUB_059750-8	5′-GCTTACACTAGTTCGATCTTGCG-3′
AFUB_051270-complement-2	5′-TCGTTACCAATGGGATCCCTTAATGCATCCCCACATGCT-3′
AFUB_059750-complement-2	5′-TCGTTACCAATGGGATCCCCTATGGAGATGCCCATCCTC-3′
Complement-3	5′-ATGCAAGAGCGGCTCATCGCCCTTCAACGCTATCACGCC-3′
Complement-4	5′-AGACATGATGGCGGTTCTCC-3′
Complement-8	5′-AAGCTGGAAGTGGGATGGCT-3′
pyrG-5	5′-CCCTTCAACGCTATCACGCC-3′
pyrG-6	5′-TCCCAGCCACAAAGGAACCT-3′
ptrA-5	5′-GGGATCCCATTGGTAACGA-3′
ptrA-6	5′-CGATGAGCCGCTCTTGCAT-3′
pyrG-F	5′-GATCTACCCCTTGGAACGCA-3′
pyrG-R	5′-GACCATCGTGGGCAATTGGT-3′
ptrA-F	5′-CATATGTAAATGGCTGTGTCCCG-3′
ptrA-R	5′-TTTAGCTTTGACCGGTGAGC-3′
pET15b-CmsB(CO)-IF-F	5′-ACAGCAGCGGCCATAGCGTGCGCCATCCATCCTCT-3′
pET15b-CmsB(CO)-IF-R	5′-CACTCGAGTGCGGCCTTATGGAATACGCGGCAGT-3′
AFUB_036480-1	5′-GAGCGCATAGATCTTGGGTAAGAC-3′
AFUB_036480-4	5′-GTGTTGCTCACCGTTCCGTTATAG-3′
AFUB_036480-7	5′-TGATGTTGTGCCTGTGTTGCTC-3′
AFUB_036480-8	5′-GGCAGCGGATCCTAACGTTATT-3′

