# Supplementary material for: Identification of Two Mannosyltransferases Contributing to Biosynthesis of the Fungal-type Galactomannan α-Core-Mannan Structure in Aspergillus fumigatus
Source: Sci Rep. 2018 Nov 16;8:16918. doi: 10.1038/s41598-018-35059-2 (PMC6240093; doi:10.1038/s41598-018-35059-2)
Supplement: Supplementary file 1 — Supplementary-materials [file 41598_2018_35059_MOESM1_ESM.pdf]

Supplementary Materials

Title: Identification of Two Mannosyltransferases Contributing to Biosynthesis of the Fungal-type Galactomannan α-Core-Mannan Structure in *Aspergillus fumigatus*

Authors: Takuya Onoue, Yutaka Tanaka, Daisuke Hagiwara, Keisuke Ekino, Akira Watanabe, Kazuyoshi Ohta, Katsuhiko Kamei, Nobuyuki Shibata, Masatoshi Goto and Takuji Oka

Supplemental Figures

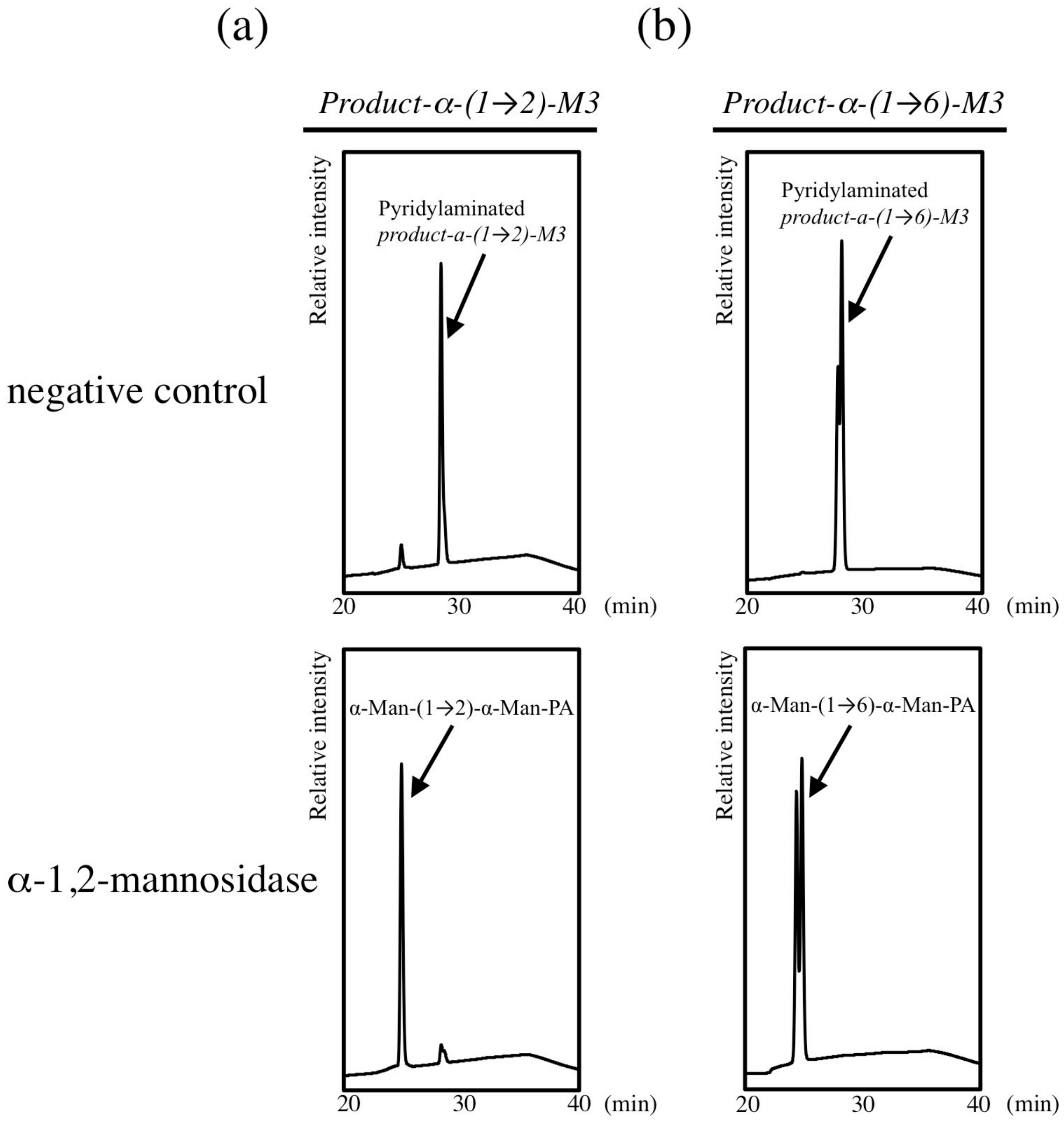

Figure S1. Determination of pyridylaminated *product-α-(1→2)-M3* (a) and *product-α-(1→6)-M3* (b) structures using substrate-specific α-1,2-mannosidase. Upper panels display chromatographs of the purified pyridylaminated *product-α-(1→2)-M3* (a) and *product-α-(1→6)-M3* (b). These products, (a) and (b), could be digested by α-1,2-mannosidase and converted to α-Man-(1→2)-α-Man-PA (a) and α-Man-(1→6)-α-Man-PA (b), respectively (lower panels).

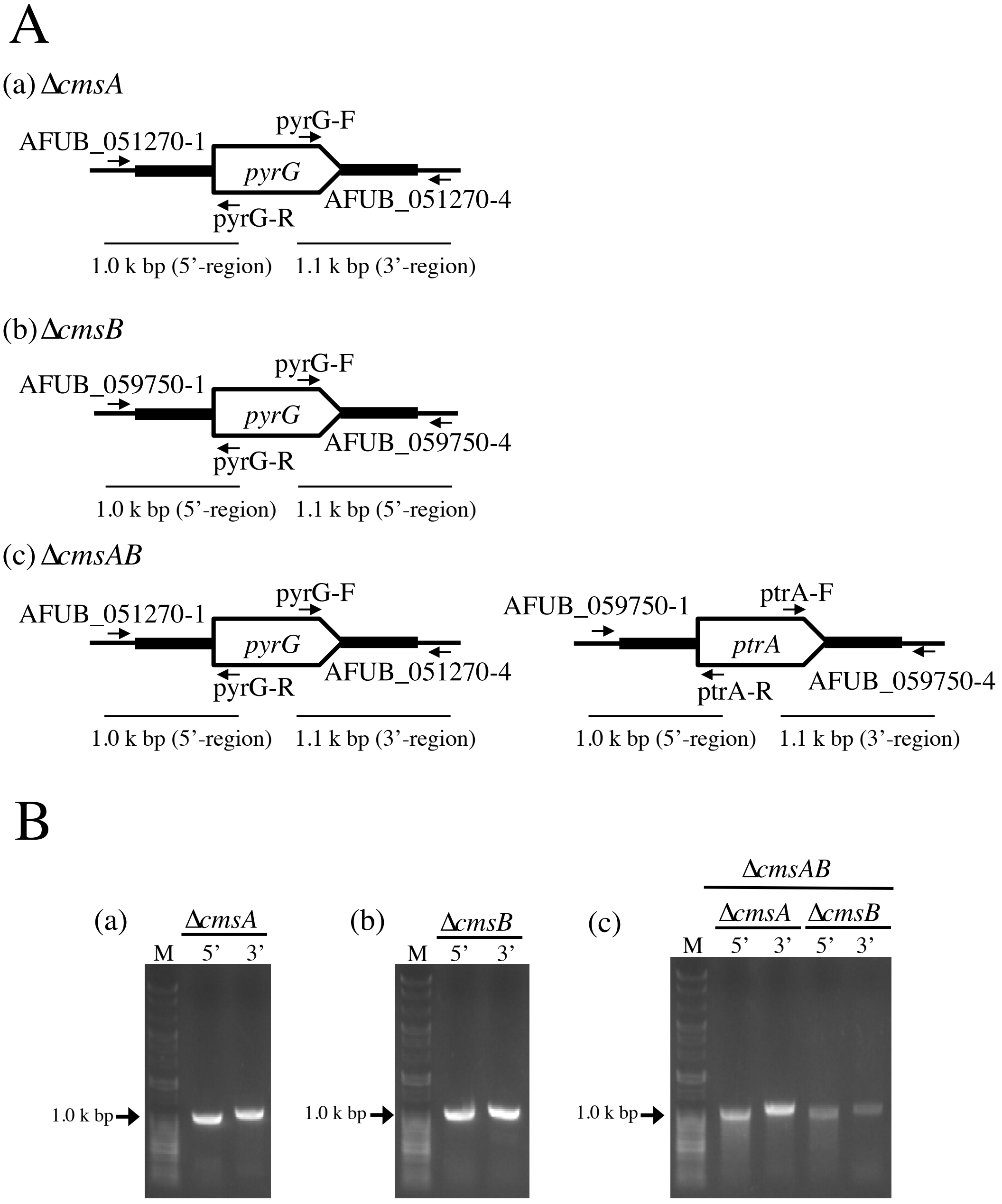

Figure S2. Construction of the core-mannan synthase disruptant strains Δ*cmsA*, Δ*cmsB*, and Δ*cmsAB*. **A.** Chromosomal maps of strains Δ*cmsA* (a), Δ*cmsB* (b), and Δ*cmsAB* (c) and primers used for confirmation. The positions of the primers are indicated by arrows. **B.** Electrophoretic analyses of products amplified by PCR using the primer pairs AFUB_051270-1/pyrG-R (5'-region) and pyrG-F/AFUB_051270-4 (3'-region) for Δ*cmsA* (a), AFUB_059750-1/pyrG-R (5'-region) and pyrG-F/AFUB_059750-4 (3'-region) for Δ*cmsB* (b), and AFUB_051270-1/pyrG-R (5'-region), pyrG-F/AFUB_051270-4 (3'-region), AFUB_059750-1/ptrA-R (5'-region), and ptrA-F/AFUB_059750-4 (3'-region) for Δ*cmsAB* (c). M: DNA size markers; Gene Ladder Wide 2 (Nippon Gene, Tokyo, Japan).

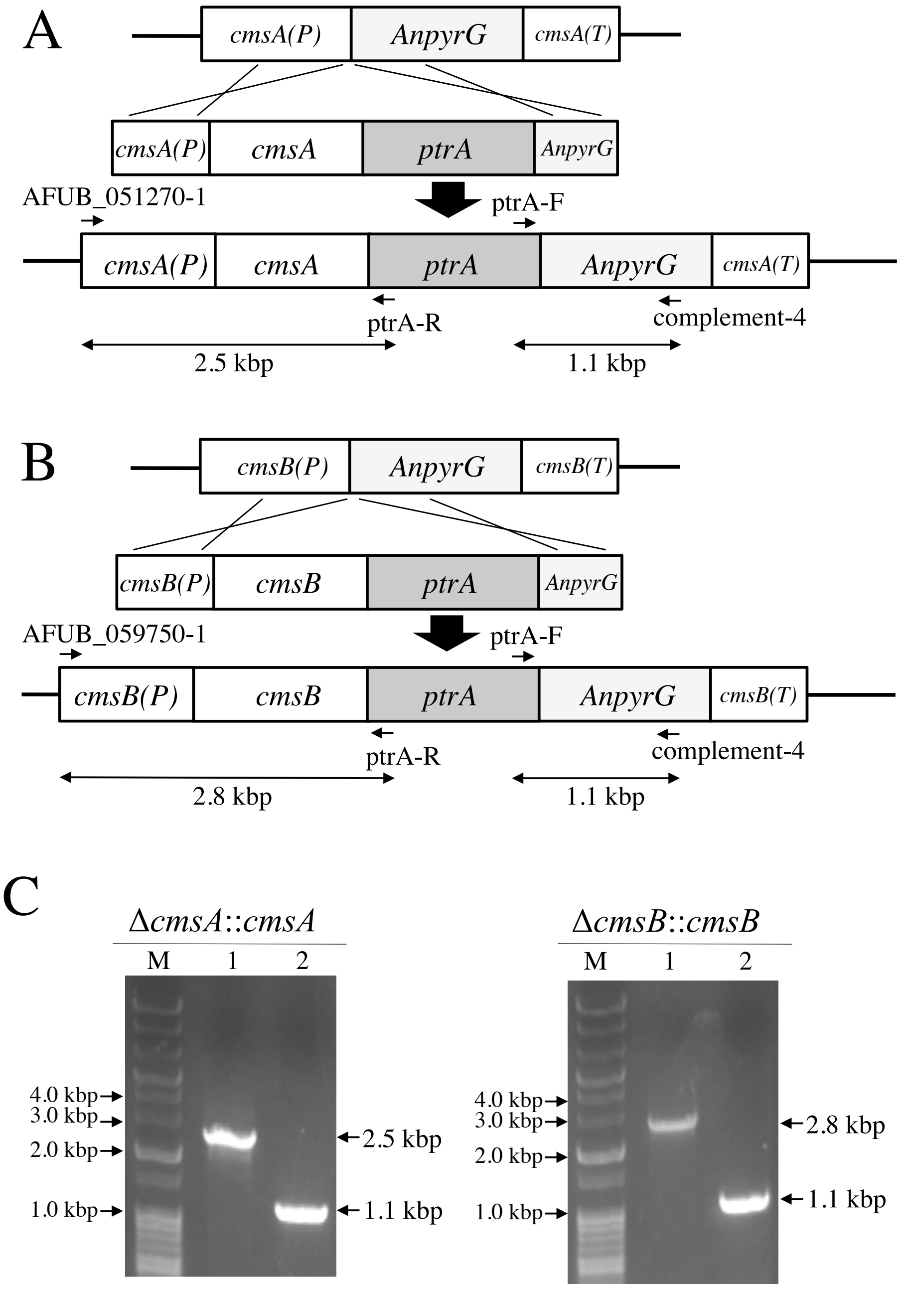

Figure S3. Construction of the Δ*cmsA* and Δ*cmsB* complementary strains Δ*cmsA*::*cmsA* and Δ*cmsB*::*cmsB*. **A.** Schematic representation of *cmsA* complementation with *cmsA*. *cmsA (P)*, *cmsA* promoter; *cmsA (T)*, *cmsA* terminator; *cmsA*, open reading frame of *cmsA*. The positions of the primers are indicated by arrows. **B.** Schematic representation of *cmsB* complementation with *cmsB*. *cmsB (P)*, *cmsB* promoter; *cmsB (T)*, *cmsB* terminator; *cmsB*, open reading frame of *cmsB*. The positions of the primers are indicated by arrows. **C.** Confirmation of correct recombination of the *cmsA* gene using PCR analysis. M, DNA size markers; Gene Ladder Wide 2; lane 1, DNA fragment (2.5 kbp) amplified using PCR and the primers AFUB_051270-1 and ptrA-R; lane 2, DNA fragment (1.1 kb) amplified using PCR and the primers ptrA-F and complement-4. **D.** Confirmation of correct recombination of the *cmsB* gene using PCR analysis. M, DNA size markers; Gene Ladder Wide 2; lane 1, DNA fragment (2.8 kbp) amplified using PCR and the primers AFUB_059750-1 and ptrA-R; lane 2, DNA fragment (1.1 kb) amplified using PCR and the primers ptrA-F and complement-4.

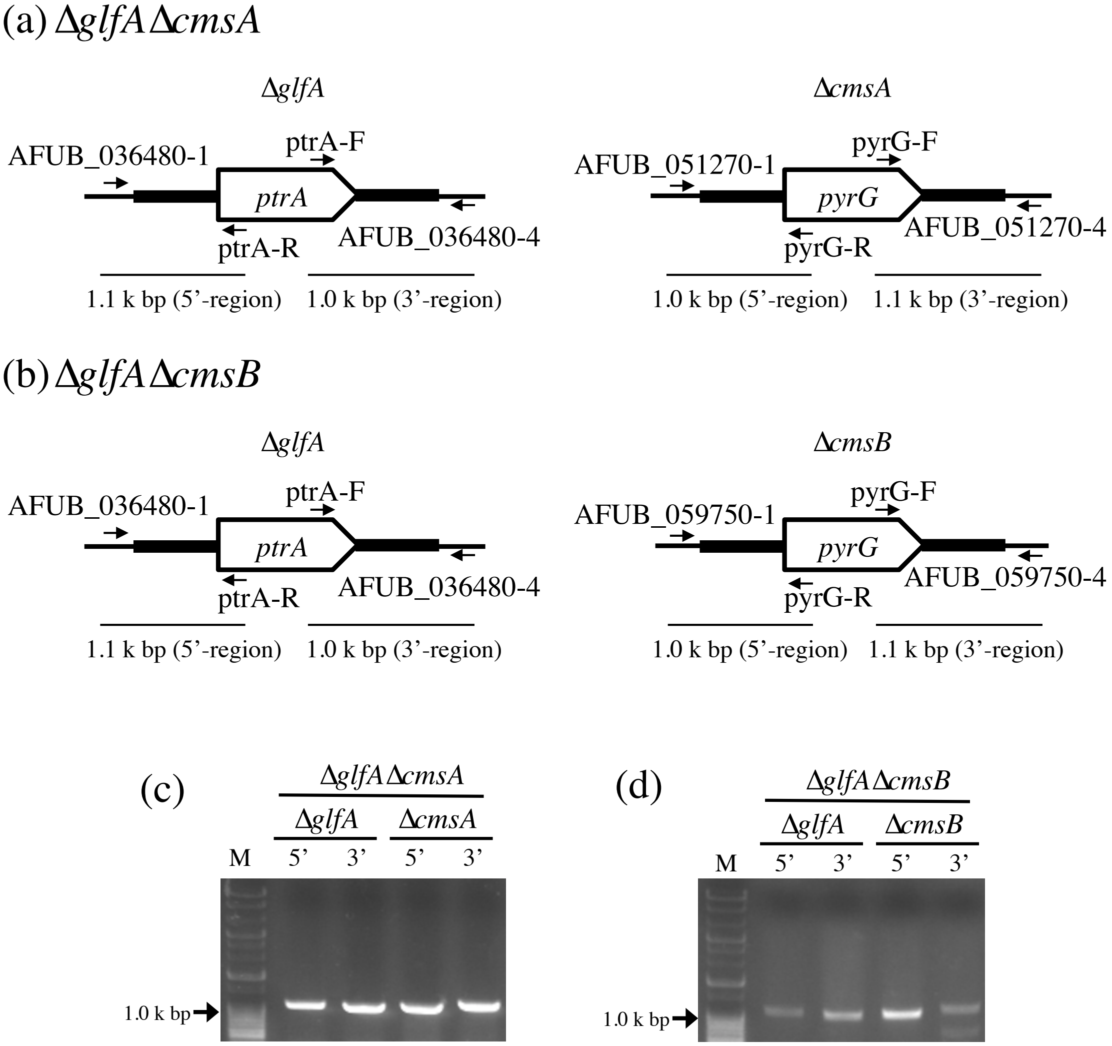

Figure S4. Construction of the Δ*glfA*Δ*cmsA* and Δ*glfA*Δ*cmsB* strains. **A.** Chromosomal maps of strains Δ*glfA*Δ*cmsA* (a) and Δ*glfA*Δ*cmsA* (b), and primers used for confirmation. The positions of the primers are indicated by arrows. **B.** Electrophoretic analyses of products amplified by PCR using the primer pairs AFUB_036480-1/ptrA-R (5’-region), ptrA-F/AFUB_036480-4 (3’-region), AFUB_051270-1/pyrG-R (5’-region), and pyrG-F/AFUB_051270-4 (3’-region), for Δ*glfA*Δ*cmsA* (c), AFUB_036480-1/ptrA-R (5’-region), ptrA-F/AFUB_036480-4 (3’-region) AFUB_059750-1/pyrG-R (5’-region), and pyrG-F/AFUB_059750-4 (3’-region), for Δ*glfA*Δ*cmsB* (d). M: DNA size markers; Gene Ladder Wide 2.

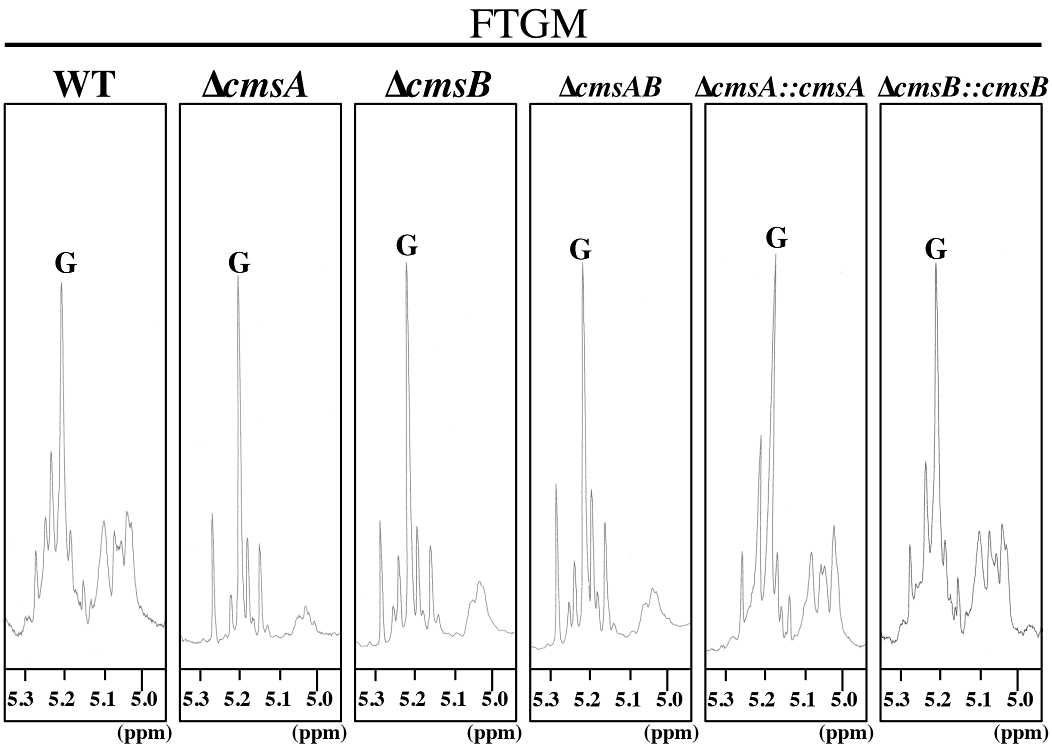

Figure S5. ^{1}H-NMR spectra of FTGM from strains A1151 (WT), Δ*cmsA*, Δ*cmsB*, Δ*cmsAB*, Δ*cmsA::cmsA*, and Δ*cmsB::cmsB*. FTGM was prepared from total GM (FTGM+OMGM) by treatment with 0.5 M $NaBH_4$/0.1 M NaOH for 24 h (β-elimination). The signal G at 5.195 ppm in the ^{1}H-NMR spectra is from H-1 at the C-1 position of the underlined Gal*f* residue in -β-Gal*f*-(1→5)-β-<u>Gal*f*</u>-(1→5)-β-Gal*f*-(1→5)-. The proton chemical shifts were referenced relative to internal acetone at δ 2.225 ppm.

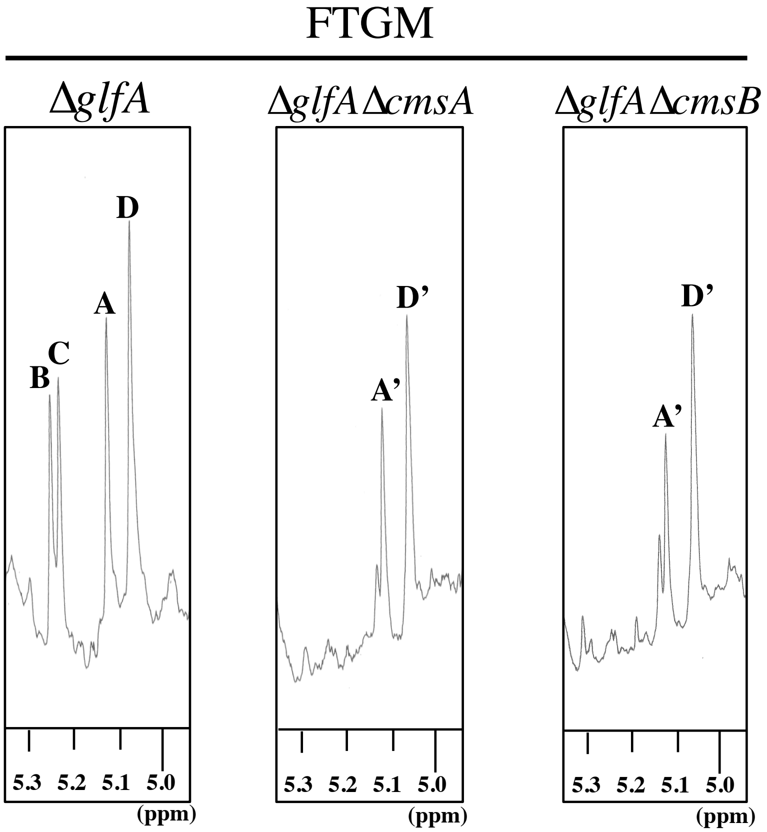

Figure S6. ^{1}H-NMR spectra of FTGM from strains Δ*glfA*, Δ*glfA*Δ*cmsA*, and Δ*glfA*Δ*cmsB*. Total GM (FTGM+OMGM) was extracted and FTGM prepared by treatment with 0.5 M $NaBH_4$/0.1 M NaOH for 24 h (β-elimination). The signals A (at 5.104 ppm), B (5.233), C (5.216), and D (5.054) in the ^{1}H-NMR spectra are from H-1 at the C-1 position of the underlined Man residue in the structures -(1→6)-α-Man-(1→2)-α-Man-(1→2)-α-Man-(1→2)-α-Man-(1→6)- (A), -(1→6)-α-Man-(1→2)-α-Man-(1→2)-α-Man-(1→2)-α-Man-(1→6)- (B), -(1→6)-α-Man-(1→2)-α-Man-(1→2)-α-Man-(1→2)-α-Man-(1→6)- (C) and -(1→6)-α-Man-(1→2)-α-Man-(1→2)-α-Man-(1→2)-α-Man-(1→6)- (D). The signals A′ and D′ at 5.1 and 5.05 ppm of the ^{1}H-NMR spectra are from the H-1 at the C-1 position of the underlined Man residue in t-Man-(1→6)-α-Man- and t-Man-(1→2)-α-Man-, respectively. The proton chemical shifts were referenced relative to internal acetone at δ 2.225 ppm.

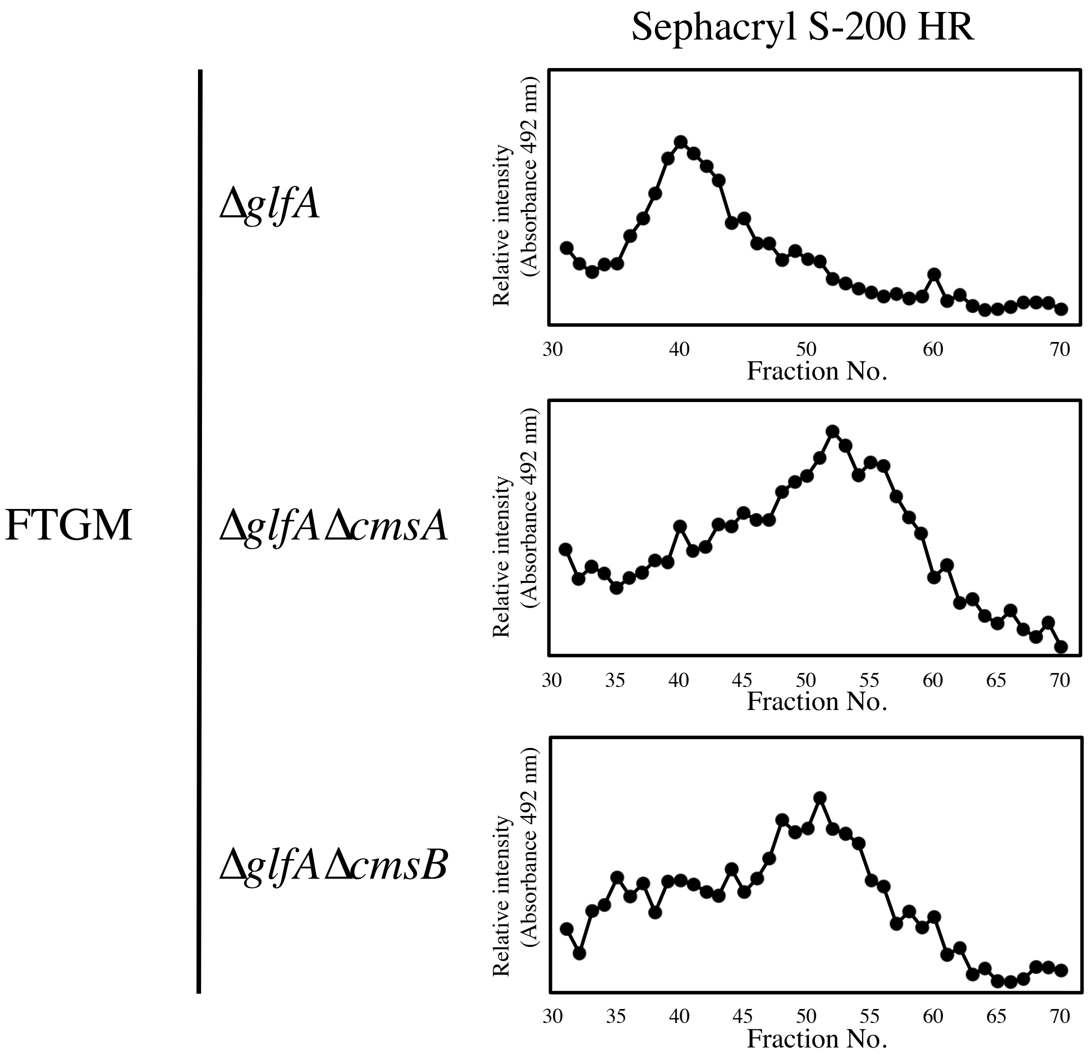

Figure S7. Gel filtration analyses of FTGM from strains Δ*glfA*, Δ*glfA*Δ*cmsA*, and Δ*glfA*Δ*cmsB*. Total GM (FTGM+OMGM) was extracted and FTGM prepared by treatment with 0.5 M $NaBH_4$/0.1 M NaOH for 24 h (β-elimination). FTGM was analyzed by gel filtration chromatogyraphy using a Sephacryl S-200 HR (1 × 75 cm) column and 0.8 M NaCl as eluent.
